# Spatial and temporal trends of the Stockholm Convention POPs in mothers’ milk — a global review

**DOI:** 10.1007/s11356-015-4080-z

**Published:** 2015-04-26

**Authors:** Johan Fång, Elisabeth Nyberg, Ulrika Winnberg, Anders Bignert, Åke Bergman

**Affiliations:** 1 Department of Environmental Science and Analytical Chemistry, Stockholm University, 106 91 Stockholm, Sweden; 2 Department of Environmental Research and Monitoring, Swedish Museum of Natural History, P.O. Box 50007, 114 18 Stockholm, Sweden; 3 Swedish Toxicology Sciences Research Center (Swetox), Forskargatan 20, 15136 Södertälje, Sweden

**Keywords:** Breast milk, Persistent organic pollutants, Stockholm Convention, DDT, Dioxin, HCH, HCB, PBDE, HBCDD

## Abstract

Persistent organic pollutants (POPs) have been of environmental and health concern for more than half a century and have their own intergovernmental regulation through the Stockholm Convention, from 2001. One major concern is the nursing child’s exposure to POPs, a concern that has led to a very large number of scientific studies on POPs in mothers’ milk. The present review is a report on the assessment on worldwide spatial distributions of POPs and of their temporal trends. The data presented herein is a compilation based on scientific publications between 1995 and 2011. It is evident that the concentrations in mothers’ milk depend on the use of pesticides and industrial chemicals defined as POPs. Polychlorinated biphenyls (PCBs) and “dioxins” are higher in the more industrialized areas, Europe and Northern America, whereas pesticides are higher in Africa and Asia and polybrominated diphenyl ethers (PBDEs) are reported in higher concentrations in the USA. POPs are consequently distributed to women in all parts of the world and are thus delivered to the nursing child. The review points out several major problems in the reporting of data, which are crucial to enable high quality comparisons. Even though the data set is large, the comparability is hampered by differences in reporting. In conclusion, much more detailed instructions are needed for reporting POPs in mothers’ milk. Temporal trend data for POPs in mothers’ milk is scarce and is of interest when studying longer time series. The only two countries with long temporal trend studies are Japan and Sweden. In most cases, the trends show decreasing concentrations of POPs in mothers’ milk. However, hexabromocyclododecane is showing increasing temporal concentration trends in both Japan and Sweden.

## Introduction

Mothers’ milk is a source of nutrients, energy, and protection for the newborn child, and it carries essential elements from the mother to the child (Kramer and Kakuma 2012). Due to the lipophilic properties of a range of anthropogenic organic pollutants, ubiquitously distributed in human food and our environment, many of these chemicals are accumulated in mothers’ milk. Accordingly, the nursing child is targeted by a vast number of undesirable pollutants (IPCS 2007; UNEP and WHO 2013). These pollutants are similar to those entering the fetus via the cord blood after transfer across the placental barrier (CDC 2013; Frederiksen et al. 2010), although there are differences in the presence of pollutants in the blood and in the mothers’ milk. Due to the high chemical and metabolic stability and toxicity of some anthropogenic chemicals as well as their ability to spread globally, and bioaccumulate, 25 chemicals have been adopted under the Stockholm Convention (SCa), known as persistent organic pollutants (POPs). Among these listed POPs, polybrominated diphenyl ethers (PBDEs) are separated into tetra-/pentaBDEs and hexa-/heptaBDEs, which actually make the POPs to 24 different entries. Six other POPs are presently under discussion for inclusion among the legacy POPs (SCb), and among these, short chain chlorinated paraffins (SCCPs) are included in the present review. The POPs reviewed herein are listed in Table 1.

**Table 1:**
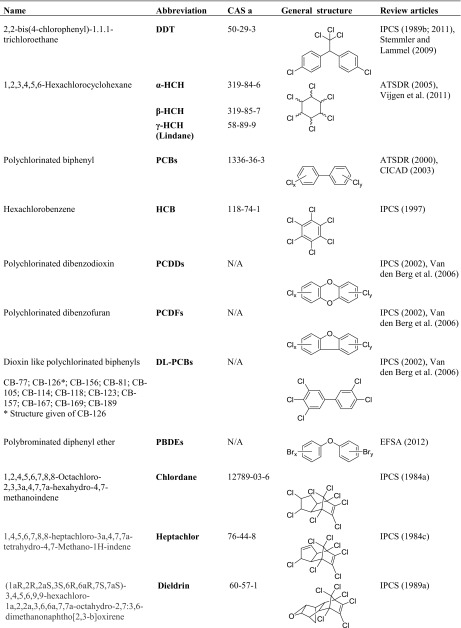

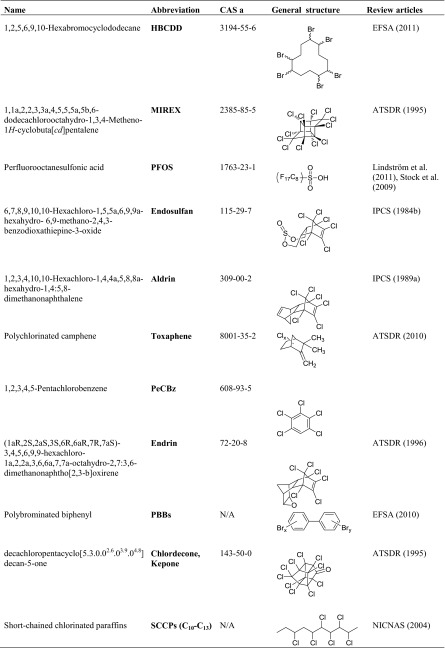
Names and abbreviations of all POPs and two suggested POPs are presented, CAS numbers are given, as well as chemical structures or general structures. Some major review documents regarding the POPs are presented under “Review articles” (the column to the far right)

The overarching toxicity of POPs is related to endocrine disruption (UNEP and WHO 2013) and/or listed as carcinogenic, mutagenic, or reprotoxic (CMRs). The toxicities of the POPs are extensively studied and will not be discussed in any detail here; instead, we prefer to refer to some of the most recent reviews on the different POPs listed in Table 1. Toxicological data for many of the POPs are often related to accidental exposures to humans or wildlife and considerable animal testing in toxicological laboratories. Some of the POPs show acute toxicity, like the “drins” (i.e., dieldrin, endrin, and aldrin). On the contrary, chronic effects have been observed for, e.g., DDT, and its transformation product dichlorodiphenyldichloroethylene (DDE), effects which were particularly emphasized in birds. The effects of many of the POPs on reproduction have been shown among wildlife species.

Accordingly, it is of interest to review the present exposure situation to POPs for nursing children worldwide, i.e., spatial exposure data as well as levels of POPs in mothers’ milk over time. The objective of this review is to summarize the concentrations of POPs in mothers’ milk during a delimited time period, 1995–2011. In addition, some recent data have been generated within the Swedish monitoring program and are included herein.

Analysis of some of the first identified POPs in mothers’ milk was published in the 1960s (Norén and Westöö 1968) and then novel POPs were added to the list (Westöö and Norén 1978). Still, as shown herein, there is limited mothers’ milk data for several of the POPs.

## Materials and methods

Names, abbreviations, and (general) structures of the POPs applied in the present review are presented in Table 1, together with reviews discussing their toxicities/ecotoxicological effects.

### Methods for data retrieval on POPs in mothers’ milk

A literature search was performed using the database Web of Science and the following search terms were used: “Human Milk,” “Breast Milk,” “Mother’s Milk,” and “Mothers Milk,” combined with the name of the substance of interest. All references found were compiled in one database and duplicates were removed.

### Methods for inclusion of data

To reduce errors due to comparison of data from different sources and to avoid presenting a historical overview, the following limits were set for inclusion in the study:

- The study must be a scientific peer-reviewed paper published 1995–2011.
- Studies must report and quantify any of the POPs listed in Table 1, in at least six subjects (donors). In the case of pooled samples, a pool should contain a minimum of six donors.
- Information about place and year of sampling are required.
- Inclusion of a time series requires a minimum of five reported data points and is only included if the report/paper includes the original values of the time series.

### Other sources of data

Data from the Swedish Environmental Monitoring Program are included herein (http://www.imm.ki.se/Datavard/aBiologiska_mätdata_-_organiska_ämnen), as well as time series data from Fång et al. (2013).

### Substance summary tables

Concentration data are presented in the substance summary tables (Tables 2, 3, 4, 5, 6, 7, 8, 9, 10, 11, 12, and 13) in the following manner:

**Table 2:** Concentrations (ng/g fat) of 4,4′-DDT and its metabolites/transformation products, 4,4′-DDE, 4,4′-DDD, and ∑DDT, in mothers’ milk, as reported in studies from around the world, 1995–2011. The concentration ratio of 4,4′-DDT/4,4′-DDE has been calculated and is presented in the table

Region	Country	4,4′-DDE			4,4′-DDD			4,4′-DDT			∑DDTs			4,4′-DDT/4,4′-DDE			Reference
		Mean	Median	GM	Mean	Median	GM	Mean	Median	GM	Mean	Median	GM	Mean	Median	GM	
Africa	Africa											980					UNEP (2011)
	Cambodia										1200; 1800	860; 1100					Kunisue et al. (2004b)
	Egypt	530^a^						73.25^a^						0.14			Saleh et al. (1996)
	Ghana	45			8			31			78			0.70			Ntow et al. (2008)
	Ghana	490															Ntow (2001)
	Ghana										230						UNEP (2009)
	Libya	120			120						420						Elafi et al. (2001)
	Mozambique	330	190		7.5	3.8		220	67		550	280		0.67	0.35		Manaca et al. (2011)
	Nigeria										860						UNEP (2009)
	Senegal										460						UNEP (2009)
	South Africa	490–3000			36–91			260–1700			800–4800			0.53–0.66			Bouwman et al. (2006)
	South Africa	4600	3600					1600	890		6300	4900		0.35	0.25		Darnerud et al. (2011)
	South Africa	70,000^a^			44,000^a^			40,000^a^			150,000^a^			0.57			Okonkwo et al. (2008)
	South Africa	2700; 6100	2400; 4600		27; 36	17; 27		2500; 4200	2000; 4500		5600; 11,000	4700; 10,000		0.69; 0.92	0.83; 0.98		Sereda et al. (2009)
	Swaziland	200									1700						Okonkwo et al. (1999)
	Tunisia	2400			280			1000			3900			0.42			Ennaceur et al. (2007)
	Tunisia	41–3100			4–320			23–1200						0.81–1.7			Ennaceur et al. (2008)
	Uganda										3000						Ejobi et al. (1998)
	Zimbabwe	1200–14,000	500–9000					250–9100	150–5400		1600–25,000	810–17,000		0.21–0.67	0.077–0.59		Chikuni et al. (1997)
Asia, Australia, and the Pacific region	Asia and the Pacific											800					UNEP (2011)
	Australia	150–870			0.06–0.45			3.6–30						0.016–0.045			Harden et al. (2007)
	Australia	310	280		0.18	0.18		11	12					0.035	0.043		Mueller et al. (2008)
	Australia																Noakes et al. (2006)
	Australia	890–1000						220–230						0.22–0.26			Quinsey et al. (1995)
	China			1300			11			65			1300			0.051	Fujii et al. (2011)
	China	1200			5.7			38			1300			0.030			Haraguchi et al. (2009)
	China	830; 2000			1.6; 6.0			40; 130			870; 2100			0.048; 0.065			Kunisue et al. (2004a)
	China		560; 720			2.2; 4.9			21; 38			580; 770			0.038; 0.050		Leng et al. (2009)
	China	1900	1800		83	94		120	140		2500	2100		0.062	0.079		Qu et al. (2010)
	China		2100; 3100	1900; 3300													Sun et al. (2005)
	China	140; 170	110; 120		3.5; 4.8	2.6; 2.7		4.5; 5.0	3.5; 3.9		150; 180	120; 130		0.029; 0.033	0.030; 0.035		Tao et al. (2008b)
	China	2800						320			3100			0.12			Tsang et al. (2011)
	China			150^a^									210^a^				Wang et al. (2008)
	China	2500; 2800						390; 700						0.16; 0.24			Wong et al. (2002)
	China	420			140			84			720			0.20			Yao et al. (2005)
	China		1300; 1500	1100; 1500													Zhao et al. (2007)
	Hong Kong										3300						Poon et al. (2005)
	India	380–1200			7.3–24			68–210			450–1500			0.18–0.22			Devanathan et al. (2009)
	India	56–56			60–66						170–180						Kumar et al. (2006)
	India	980; 1000						1200; 1400			2900; 3200			1.2; 1.3			Mishra and Sharma (2011)
	India																Sanghi et al. (2003)
	India	9500^a^			75^a^			2750^a^			14,000^a^			0.29			Siddiqui et al. (2002)
	India	16,800^a^			5200^a^			3950^a^			32000^a^			0.24			Nair et al. (1996)
	Indonesia	400						90						0.23			Burke et al. (2003)
	Indonesia	600–1100	140–860		1.2–2.8	0.44–1.2		32–180	8.4–17		640–1300	160–910		0.029–0.16	0.01–0.071		Sudaryanto et al. (2006)
	Indonesia	940	670		1.3	0.47		39	14		990	670		0.041	0.021		Sudaryanto et al. (2008a)
	Iran	1300–2900	490–1100		2.0–34			170–480	6.0–270		1600–3600	700–1400		0.13–0.32	0.0070–0.33		Behrooz et al. (2009)
	Iran	1700						300			2200			0.18			Cok et al. (1999)
	Japan			110			3.2			6.7			120			0.062	Fujii et al. (2011)
	Japan	92–250			1.1–1.7			4.0–6.9			97–260			0.028–0.43			Haraguchi et al. (2009)
	Japan	270						17.8						0.066			Konishi et al. (2001)
	Japan	260			0.78			11			280			0.042			Kunisue et al. (2006)
	Japan		72														Miyake et al. (2011)
	Japan							92^a^									Nagayama et al. (2007a)
	Japan											290					Nagayama et al. (2007b)
	Jordan	260						80						0.30			Alawi et al. (2006)
	Jordan	2000-9400			1960			4400			9700			2.3			Nasir et al. (1998)
	Kazakhstan	2000	1500														Lutter et al. (1998)
	Kazakhstan	1800–3300						220–460						0.12–0.31			Hooper et al. (1997)
	Korea			120			4.2			140			140			1.2	Fujii et al. (2011)
	Korea	170			2			10			180			0.059			Haraguchi et al. (2009)
	Korea										160						UNEP (2009)
	Kuwait	830			4.2			12			850			0.015			Saeed et al. (2000)
	Malaysia	1600			3			46			1600			0.029			Sudaryanto et al. (2005)
	Philippines	56–160	30–65		0.57–1.2	0.33–0.44		5.0–7.1	3.6–5.1		60–170	38–70		0.045–0.089	0.075–0.12		Malarvannan et al. (2009)
	Russia	530; 600			2.2; 3.6			50; 52			580; 660			0.083; 0.098			Tsydenova et al. (2007)
	Saudi Arabia	490; 540			6.0; 23			71; 110			570; 680			0.14; 0.20			Al-Saleh et al. (2002)
	Saudi Arabia	240–1800	200; 260		2.0–360			37–1200	62; 88		280–3300	260; 400		0.10–0.67	0.32; 0.34		Al-Saleh et al. (2003)
	Taiwan	310						23			330			0.074			Chao et al. (2006)
	Thailand	7200^a^	4200^a^		200^a^	170^a^		2300^a^	1700^a^		9800^a^	5200^a^		0.32	0.41		Stuetz et al. (2001)
	Turkey	1900; 2000						72; 140			2200; 2700			0.036; 0.076			Cok et al. (1997)
	Turkey	2100						110			2600			0.053			Cok et al. (2005)
	Turkey	1000						260			1400			0.24			Cok et al. (2011)
	Turkey		1500		1.8	1.5			65			1600			0.043		Erdogrul et al. (2004)
	Turkey	6.8	3.6		10	5.6		20	11		37	20		3.0	3.1		Ozcan et al. (2011)
	Uzbekistan		870						70						0.080		Ataniyazova et al. (2001)
	Vietnam	1200			9.4			56			1200			0.047			Haraguchi et al. (2009)
	Vietnam	1900			7.0; 11			170; 260			2100; 2300			0.089; 0.13			Minh et al. (2004)
	Vietnam		220; 720			1.8; 2.1			14; 20			240; 750			0.028; 0.064		Nguyen et al. (2010)
Europe	Belgium	120															Colles et al. (2008)
	Central and Eastern Europe											380					UNEP (2011)
	Croatia	27	22		8.8	4.7		7.1	2.1					0.26	0.10		Frkovic et al. (1996)
	Croatia		230; 260			2; 5			14; 19						0.060; 0.075		Kozul and Romanic (2010)
	Croatia	250–490															Krauthacker et al. (1998)
	Croatia		100–380						13; 170						0.12; 0.60		Krauthacker et al. (2009)
	Czech Republic	920; 1000	820; 860					81; 86	60; 74					0.080; 0.093	0.073; 0.086		Cajka and Hajslova (2003)
	Czech Republic	260–840	170–680					24–98	14–90		280–920	240–730		0.044–0.14	0.042–0.14		Cerna et al. (2010)
	Czech Republic										830-1300						(Schoula et al. 1996)
	Denmark		59; 130			0.31; 0.36			3.4; 5.7			63; 140			0.042; 0.058		Shen et al. (2008)
	Denmark and Finland		90			8.7			4.3			130			0.048		Damgaard et al. (2006)
	England																Thomas et al. (2006)
	Finland	77; 140			0.41; 0.48			4.4; 7.1			82; 140			0.052; 0.056			Shen et al. (2007)
	France		80														Brucker-Davis et al. (2010)
	Germany	160	87						4		180	100			0.046		Raab et al. (2008)
	Germany							240	200								Schade and Heinzow (1998)
	Germany										360; 380						Schlaud et al. (1995)
	Germany	550															Skopp et al. (2002)
	Germany							130–170	81–110								Zietz et al. (2008)
	Greece	720			15			66			800			0.091			Schinas et al. (2000)
	Italy	210–510						9.4–44						0.045–0.10			Abballe et al. (2008)
	Latvia	150–240						13–22						0.069–0.12			Bake et al. (2007)
	Norway	93–110	93–110		0.2–0.3	0.2–0.3		7.2–10	7.5		100–120	100–120		0.064–0.11	0.63–0.10		Polder et al. (2008b)
	Norway	53; 140	41; 61														Polder et al. (2009)
	Poland	500–690^a^			12–30^a^			62–140^a^						0.15–0.24			Czaja et al. (1997b)
	Poland	820	630					51	42		870	680		0.062	0.066		Jaraczewska et al. (2006)
	Romania	350			81			34			700			0.10			Cioroiu et al. (2010)
	Romania	1700^a^	1200^a^		44^a^	29^a^		310^a^	190^a^		2000^a^	1400^a^		0.19	0.16		Covaci et al. (2001)
	Russia	800; 810	680; 710		3; 5	3; 3		91; 220	78; 160		900; 1000	720; 920		0.11; 0.27	0.12; 0.22		Polder et al. (2008a)
	Russia	890; 1300	900; 1200		4.1; 8.2	3.4; 6.5					1100; 1500	1000; 1200					Polder et al. (1998)
	Slovak Republic	9.0–1300	1.0–430					7.0–180	5.0–110					0.024–1.2	0.15–8.0		Veningerova et al. (2001)
	Slovak Republic	630; 660	540; 540					33; 36	26; 27					0.052; 0.055	0.048; 0.050		Yu et al. (2007)
	Spain	220			1.2			12			240			0.052			Bordajandi et al. (2008)
	Spain	390^a^			4.3^a^			12^a^						0.030			Pico et al. (1995)
	Spain		800; 1000														Ribas-Fito et al. (2005)
	Sweden	100	72					5.7	3.7					0.056	0.051		Aune et al. (2002)
	Sweden	58; 160	52					4.0; 7.0	3.7					0.044; 0.069	0.071		Bergman et al. (2010)
	Sweden	120	78					6.0	5.2					0.053	0.067		Darnerud (2001)
	Sweden		110														Darnerud et al. (2010)
	Sweden		46–72						65						0.98		Glynn et al. (2011)
	Sweden	70	59					4.5	4.2		75			0.065	0.072		Lignell et al. (2003)
	Sweden	270	100					9.1	6.1					0.033	0.060		Lignell et al. (2004)
	Sweden	230						22						0.10			Lundén and Norén (1998)
	Sweden	250						32						0.13			Norén et al. (1996)
	Sweden	84–190	82					1.7–3.2	1.3					0.016–0.022	0.016		Athanasiadou and Bergman (2008)
	Netherlands	550							40								Albers et al. (1996)
	Ukraine		2300; 2800						320; 340						0.12; 0.14		Gladen et al. (1999)
	Ukraine		2500						340						0.14		Gladen et al. (2003)
	UK	430	280					40	25					0.093	0.088		Harris et al. (1999)
	UK		150	150		0.3	0.3		6.2	6.2		160	160		0.041	0.04	Kalantzi et al. (2004)
	Western Europe and other States											82					UNEP (2011)
	Yugoslavia	330; 460^a^	280; 360^a^					12; 360^a^	290^a^					0.038; 0.79	0.79		Vukavic et al. (2003)
The Americas	Antigua and Barbuda										190						UNEP (2009)
	Brazil	1500			6			180			1700			0.12			Paumgartten et al. (2000)
	Brazil	29			72			360			460			12			Azeredo et al. (2008)
	Canada	340															Dewailly et al. (1996)
	Canada			960													Dewailly et al. (2000)
	Canada	440	320					24	21					0.055	0.065		Newsome and Ryan (1999)
	Canada	220	93–150^a^					22	12–18^a^					0.10	0.12–0.14		Newsome et al. (1995)
	Canada and USA			180													Fitzgerald et al. (2001)
	Chile										210						UNEP (2009)
	GROLAC^b^											200					UNEP (2011)
	Mexico		2500–1300			140; 250											Elvia et al. (2000)
	Mexico	5300			88			2100			7800			0.40			Pardio et al. (1998)
	Mexico	3000						210			3100			0.069			Rodas-Ortiz et al. (2008)
	Mexico	590						160			900			0.27			Torres-Arreola et al. (1999)
	Mexico	3900			70			1600			5700			0.40			Waliszewski et al. (1998)
	Mexico	4000; 4800			2.0; 5.0			650; 900			4700; 5700			0.16; 0.19			Waliszewski et al. (1999b)
	Nicaragua	2800						130						0.046			Romero et al. (2000)
	Uruguay										130						UNEP (2009)
	USA			190													Fitzgerald et al. (2001)
	USA	270	260														Greizerstein et al. (1999)
	USA	53	35		3.1	2.7		6.7			65	41		0.13			Johnson-Restrepo et al. (2007)
	USA	220															Kostyniak et al. (1999)
	USA	120						5						0.041			Pan et al. (2010)
	USA	110; 440^a^	79; 87^a^					3.1; 9.4^a^	2.6; 2.7^a^		120; 460^a^	83; 91^a^		0.021; 0.027	0.029; 0.033		Weldon et al. (2011)
	Venezuela										280–1000^a^						Brunetto et al. (1996)

When concentrations from more than one sampling site in the same country and study have been reported, the concentrations are given as two values separated by a semicolon “;” (two concentrations) or a dash “–” (for more than two concentrations)

^a^Recalculated from fresh weight, assuming 4 % fat content

^b^Group of Latin America and Caribbean countries

**Table 3:** Concentrations (ng/g fat) of CB-153, ∑ 6 indicator PCBs, and ∑PCBs, in mothers’ milk, as reported in studies from around the world, 1995–2011

Region	Country	CB-153		∑ 6 indicator PCBs^a^		∑PCB			Reference
		Mean	Median	Mean	Median	Mean	Median	GM	
Africa	Africa				31				UNEP (2011)
	Ghana	6.4–22	5.4–19			30–82	26–72		Asante et al. (2011)
	South Africa	2.6	2			10	8.4		Darnerud et al. (2011)
	Tunisia	18–120		95–660		110–750			Ennaceur et al. (2008)
	Zimbabwe					2.8–60			Chikuni et al. (1997)
Asia, Australia, and the Pacific region	Asia and the Pacific				15				UNEP (2011)
	Australia					160–480			Quinsey et al. (1995)
	Cambodia					20–29	13–24		Kunisue et al. (2004a)
	China					28–42			Kunisue et al. (2004a)
	China					74			Poon et al. (2005)
	China	14				49			Tsang et al. (2011)
	China					33; 42			Wong et al. (2002)
	China					9.5			Xing et al. (2009)
	China	0.49–16		2.4–29					Zhang et al. (2011)
	China		13.28				210	210	Zhao et al. (2007)
	India					23–40			Devanathan et al. (2009)
	Indonesia					21–33	17–27		Sudaryanto et al. (2006)
	Iran	200–250	46–150	990–1900	130–1000				Behrooz et al. (2009)
	Japan					120	110		Kawashiro et al. (2008)
	Japan					200			Konishi et al. (2001)
	Japan					120			Kunisue et al. (2006)
	Japan					120	100		Nakamura et al. (2008)
	Japan						110		Nagayama et al. (2007a)
	Japan	19	18	39	36	73	67		Todaka et al. (2011)
	Jordan	25				42			Alawi et al. (2006)
	Kazakhstan			100–430		220–820			Hooper et al. (1997)
	Kazakhstan			100–350		410			Lutter et al. (1998)
	Kazakhstan	65	50	180	130	370	290		She et al. (1998)
	Malaysia					14; 80			Sudaryanto et al. (2005)
	Russia					160–240			Tsydenova et al. (2007)
	Taiwan			55			54		Wang et al. (2004)
	Philippines					50–70	40–60		Malarvannan et al. (2009)
	Turkey	11		26		27			Cok et al. (2011)
	Turkey	110		190		210			Cok et al. (2003)
	Turkey	3.4–11		11–19		18–36			Cok et al. (2009)
	Turkey	8.5^b^	8.2^b^			27^b^	28^b^		Erdogrul et al. (2004)
	Turkey	12	6.5	100	69				Ozcan et al. (2011)
	Vietnam	11–43				56–150			Haraguchi et al. (2009)
	Vietnam					74; 79			Minh et al. (2004)
	Vietnam		5.7; 8.2			33; 46	24; 33		Tue et al. (2010)
	Vietnam		4.7; 8.1		14; 22		33; 47		Nguyen et al. (2010)
Europe	Belgium	43		97		110			Colles et al. (2008)
	Central and Eastern Europe				47				UNEP (2011)
	Croatia		39; 42		110; 110		120; 130		Kozul and Romanic (2010)
	Croatia						210		Krauthacker et al. (1998)
	Croatia		29				120		Krauthacker et al. (2009)
	Croatia		10		110		140		Zubcic and Krauthacker (2004)
	Czech Republic	220; 420		530; 1100		620; 1200			Bencko et al. (1998)
	Czech Republic	260; 300	220; 260			940; 1100	780; 900		Cajka and Hajslova (2003)
	Czech Republic	93–900	98–650	300–2000	310–1500	490–4900	480–3400		Cerna et al. (2010)
	Czech Republic	270–480				860–1100			Schoula et al. (1996)
	Finland	88; 110				350; 440			Vartiainen et al. (1997)
	France		59				170		Brucker-Davis et al. (2010)
	Germany					550	500		Schade and Heinzow (1998)
	Germany					540; 1300			Schlaud et al. (1995)
	Germany	140				310			Skopp et al. (2002)
	Germany	90				210			Wittsiepe et al. (2007)
	Germany					200	180		Zietz et al. (2008)
	Italy	54		120		200			Alivernini et al. (2011)
	Italy	110	110			280	280		Riva et al. (2004)
	Italy	22–38		50–92		82–130			Ulaszewska et al. (2011)
	Latvia	16–24				110–170			Bake et al. (2007)
	Lithuania	130–160		290–360		400–480			Becher et al. (1995)
	Netherlands		77		240		270		Albers et al. (1996)
	Netherlands	120–140		300–350		360–410			van den Berg et al. (1995)
	Norway	130–140		270; 300		330–380			Becher et al. (1995)
	Norway						99		Eggesbo et al. (2006)
	Norway	44–53	52	100–120	100–120	160–180	160–200		Polder et al. (2008b)
	Norway					110; 120	100; 110		Polder et al. (2009)
	Poland					190–550^b^			Czaja et al. (1997a)
	Poland	40	35			150	130		Jaraczewska et al. (2006)
	Poland					170–350			Pietrzak-Fiecko et al. (2005)
	Poland	30; 38		56; 77		82;97			Skrbic et al. (2010)
	Romania	58^b^	37^b^			9.7	6.5		Covaci et al. (2001)
	Russia	120; 130	110; 120	30–230	20–210	300–350	290; 330		Polder et al. (1998)
	Russia	50; 90	50; 50	120; 200	100; 180	190; 350	180; 320		Polder et al. (2008a)
	Serbia	26^b^	11^b^	76^b^	27^b^	81^b^	31^b^		Vukavic et al. (2008)
	Slovak Republic	230–480		540–1200		590–1300			Petrik et al. (2001)
	Slovak Republic	200; 200	180; 180			600; 650	500; 540		Yu et al. (2007)
	Spain					280			Schuhmacher et al. (2009)
	Sweden	42–70	51	87–140	100	120–180	140		Athanasiadou and Bergman (2008)
	Sweden	100				240			Atuma et al. (1998)
	Sweden	51	48			140	130		Aune et al. (2002)
	Sweden	29; 30	27			99; 100	98		Bergman et al. (2010)
	Sweden	56	55	110	110	148	144		Darnerud (2001)
	Sweden		64						Darnerud et al. (2010)
	Sweden	74	69	150	136	170	160		Glynn et al. (2001)
	Sweden		31–48				80–120		Glynn et al. (2011)
	Sweden	61				190			Guvenius et al. (2003)
	Sweden	47	43			120	110		Lignell et al. (2003)
	Sweden	62	57			150	140		Lignell et al. (2004)
	Sweden	58	52			140	120		Lignell et al. (2009b)
	Sweden	58				110			Lignell et al. (2011)
	Sweden	96		200		380			Lundén and Norén (1998)
	Sweden	96				410			Norén et al. (1996)
	Switzerland	36	28			290	240		Zehringer and Herrmann (2001)
	Ukraine						600		Gladen et al. (2003)
	Ukraine						490; 680		Gladen et al. (1999)
	UK		49				180	150	Kalantzi et al. (2004)
	Western Europe and other States				79				UNEP (2011)
	Former Yugoslavia					11; 20	4.9; 8.2		Vukavic et al. (2003)
The Americas	Brazil	37				150			Paumgartten et al. (2000)
	Canada	54				130			Dewailly et al. (1996)
	Canada							620	Dewailly et al. (2000)
	Canada	38	33.4			240	210		Newsome et al. (1995)
	Canada					250	240		Newsome and Ryan (1999)
	Canada and USA	50				220			Fitzgerald et al. (1998)
	GROLAC^c^				28				UNEP (2011)
	Mexico	110				1500			Rodas-Ortiz et al. (2008)
	USA	33				120			Fitzgerald et al. (1998)
	USA	69	65			300	280		Greizerstein et al. (1999)
	USA			57^d^		270			Kostyniak et al. (1999)
	USA	17					77		Pan et al. (2010)
	USA					91			Park et al. (2011)
	USA	1.5; 9.0^b^	1.1; 6.0^b^			22; 29^b^	19; 20^b^		Weldon et al. (2011)

When concentrations from more than one sampling site in the same country and study have been reported, the concentrations are given as two values separated by a semicolon “;” (two concentrations) or a dash “–” (for more than two concentrations)

^a^Sum of CB-28, CB-52, CB-101, CB-138, CB-153, and CB-180

^b^Recalculated from fresh weight, assuming 4 % fat content

^c^Group of Latin America and Caribbean countries

^d^Reported as the sum of CB-105, CB-132, and CB-153

**Table 4:** Concentrations (ng/g fat) of hexachlorobenzene (HCB) and the three HCH isomers, α-HCH, β-HCH, and γ-HCH, in mothers’ milk, as reported in studies from around the world, 1995–2011, are presented. Also the ∑HCH data are presented, giving data as reported in the studies referred to

Region	Country	HCB			α-HCH			β-HCH			γ-HCH			δ-HCH			∑HCHs			Reference
		Mean	Median	Geo. mean	Mean	Median	Geo. mean	Mean	Median	Geo. mean	Mean	Median	Geo. mean	Mean	Median	Geo. mean	Mean	Median	Geo. mean	
Africa	Africa		2.8																	UNEP (2011)
	Egypt										210^a^						210			Saleh et al. (1996)
	Ghana	4.9			190			14									46			Darko and Acquaah (2008)
	Ghana	40																		Ntow (2001)
	Ghana	2.5–14															3.5–65			UNEP (2009)
	Libya				140			240			120						500			Elafi et al. (2001)
	Nigeria	5.0															29			UNEP (2009)
	Senegal	3.7															65			UNEP (2009)
	South Africa	1.9	1.8														12	4.3		Darnerud et al. (2011)
	Swaziland																			Okonkwo et al. (1999)
	Tunisia	0.38–290						16–110			3–70						26–130			Ennaceur et al. (2008)
	Tunisia	260						50									67			Ennaceur et al. (2007)
	Uganda																			Ejobi et al. (1998)
Asia, Australia, and the Pacific region	Asia and the Pacific		1.25																	UNEP (2011)
	Australia	6.6–76	14.3		0.03–0.18	0.047		7.6–660	21		0.08–.47	0.22					7.7–660	21		Harden et al. (2007)
	Australia	30	19		0.18	0.13		24	27		0.2	0.2					24	27		Mueller et al. (2008)
	Australia																			Noakes et al. (2006)
	Australia	370–460			61–85			200–550			100–130			100–130			500–900			Quinsey et al. (1995)
	Australia	51	35					9.8									9.8			Khanjani and Sim (2006)
	Cambodia	1.6; 1.8	1.4; 1.5														4.8; 5.6	3.5; 3.6		Kunisue et al. (2004a)
	China	56; 81			4.7; 5.0			550; 1400			0.93; 1.3						550; 1400			Kunisue et al. (2004a)
	China		19; 48			3.7			240; 630			1.8; 1.8						240; 640		Leng et al. (2009)
	China				5.3	3.9		55	42		4.5	2.9		4.3	1.5		80	55		Qu et al. (2010)
	China		88; 100	85; 98					10; 180	21; 110										Sun et al. (2005)
	China							950; 1100												Wong et al. (2002)
	China				64			49.5			55.6						170			Yao et al. (2005)
	China				0.80; 1.4		0.67; 1.0	310; 360		210; 280	2.3		2.4	0.91		0.72	310; 360		210; 290	Yu et al. (2009)
	China		38; 48	33; 47		25; 76	18; 53		160; 210	200; 210		6.0; 17	8.0; 14					190; 350	260; 270	Zhao et al. (2007)
	China	33															230			Zhou et al. (2011)
	Hong Kong																1000			Poon et al. (2005)
	Hong Kong and China	22			0.6			940			1.8						940			Hedley et al. (2010)
	India				1800			8800			2300						13,000			Banerjee et al. (1997)
	India	1.7–4.4			4.6–9.1			210–680			1.1–82						220–670			Devanathan et al. (2009)
	India				32–37			39–43			51–54						120–130			Kumar et al. (2006)
	India				640; 830			1000; 1100			600; 620			77; 170			2700; 2300			Mishra and Sharma (2011)
	India				1100^a^			5000^a^			2100^a^						8200^a^			Nair et al. (1996)
	India							1600^a^			900^a^			100^a^			2600^a^			Sanghi et al. (2003)
	India				1800^a^			16,000^a^			1300^a^			2300^a^			22,000^a^			Siddiqui et al. (2002)
	Indonesia	60						90									90			Burke et al. (2003)
	Indonesia	1.8–2.3	1.6–2.3		0.02–0.22	0.08; 0.18		6.6–31	5.4–9.0		0.26–1.2	0.12; 0.22					7–30	5.5–8.3		Sudaryanto et al. (2006)
	Indonesia	2.1	1.8		0.18			12	6.3		0.68	0.08					12	6.8		Sudaryanto et al. (2008a)
	Iran	630–1500	280–820		880–1700	420–1300		1600–4000	910–15,000		126–460	70–240					2600–5700	1400–17,000		Behrooz et al. (2009)
	Iran	61			22			400			180						600			Cok et al. (1999)
	Japan	14						210									210			Konishi et al. (2001)
	Japan	13			0.48			92									92			Kunisue et al. (2006)
	Japan		7						28									28		Miyake et al. (2011)
	Japan	0.18															1.2			Nagayama et al. (2007a)
	Japan								330									330		Nagayama et al. (2007b)
	Japan	34						63									63			Saito et al. (2005)
	Jordan	61			82			390			39						510			Alawi et al. (2006)
	Jordan	350			180						710						890			Nasir et al. (1998)
	Kazakhstan	73–97	71–80					1700–2300	1400–1800											Lutter et al. (1998)
	Kazakhstan	52–180			41–97			1600–3500									1600–3500			Hooper et al. (1997)
	Korea	7.7															20			UNEP (2009)
	Kuwait				0.69			3.6			1.1						5.4			Saeed et al. (2000)
	Malaysia	11			1			230			1.3						230			Sudaryanto et al. (2005)
	Philippines	1.7–2.5	1.5–1.9		0.24–0.29	0.17–0.25		3.2–4.9	2.7–4.0		0.15–0.34	0.14–0.16					3.6–5.5	3.1–4.3		Malarvannan et al. (2009)
	Taiwan							1.9			1.5						3.4			Chao et al. (2006)
	Thailand	130^a^	140^a^								90^a^									Stuetz et al. (2001)
	Turkey	44–58			50–67			360–420			16–17						440–480			Cok et al. (1997)
	Turkey	39			1			150			8						160			Cok et al. (2011)
	Turkey	73			27			280			14						340			Cok et al. (2005)
	Turkey		20						150			3						150		Erdogrul et al. (2004)
	Turkey				5.9	2.5		5.90	14		2.8	10		8.04	5.01		23	32		Ozcan et al. (2011)
	Uzbekistan		28			76			1100			8						1200		Ataniyazova et al. (2001)
	Vietnam	7.4–86						49–570												Haraguchi et al. (2009)
	Vietnam	2.5; 3.9						14; 58												Minh et al. (2004)
	Vietnam		1.8; 3.0			0.20			5.1; 18			0.055						5.8; 18		Nguyen et al. (2010)
Europe	Belgium	16.2																		Colles et al. (2008)
	Central and Eastern Europe		3.1																	UNEP (2011)
	Croatia	4.2	4.0								1.1						1.1			Frkovic et al. (1996)
	Croatia		7.4–12			1.5–2.4			19–20			15–18						36–41		Romanic and Krauthacker (2006)
	Croatia		11–31						24–39									26–40		Krauthacker et al. (1998)
	Croatia		5–13			2			12–54			7–14						24–62		Krauthacker et al. (2009)
	Czech Republic	320; 420	250; 370					56; 64	55; 57								56; 64	55; 57		Cajka and Hajslova (2003)
	Czech Republic	119–748	92–357					22–36	20–27											Cerna et al. (2010)
	Czech Republic	480–640						71–80												Schoula et al. (1996)
	Denmark	12			0.51			18			0.74			0.06			740	740		Shen et al. (2007)
	Denmark		12			0.26			17			0.65			0.04			18		Shen et al. (2008)
	Finland	8.4			0.19			12			0.61			0.04			860	660		Shen et al. (2007)
	Finland		8.0			0.16			11			0.4			0.03			12		Shen et al. (2007)
	Finland and Denmark		10			0.19			13									13		Damgaard et al. (2006)
	France		23																	Brucker-Davis et al. (2010)
	Germany	27	21					17	8								17	8		Raab et al. (2008)
	Germany	80	70					40	40								40	40		Schade and Heinzow (1998)
	Germany	150; 220						45; 59			12; 16						61; 71			Schlaud et al. (1995)
	Germany	27; 38	23; 31					21; 27	12; 17		1.7; 3.7	3					22–30	12–20		Zietz et al. (2008)
	Greece				6.0			15.51			7.0			6.8			59			Schinas et al. (2000)
	Italy	38–70																		Abballe et al. (2008)
	Latvia	19–32			0.18–0.27			42–90			0.06–0.34						42–90			Bake et al. (2007)
	Norway	12	12																	Eggesbo et al. (2009)
	Norway	17–19	18–20		0.2–0.2	0.2–0.2		10–16	9.2–14		0.3–0.7	0.3–0.5					10–17	9.7–14		Polder et al. (2008b)
	Norway	11; 15	11; 13					5.4; 18	4.7; 8.1											Polder et al. (2009)
	Poland	33–55^a^			5.0–20^a^			35–100^a^			5–12^a^						45–130^a^			Czaja et al. (1997b)
	Poland	32	29					13.3	11		0.8						14	11		Jaraczewska et al. (2006)
	Romania	20			43			27.5			75			45			190			Cioroiu et al. (2010)
	Romania	16^a^	14^a^		17^a^	15^a^		480^a^	440^a^		35^a^	15^a^					520^a^	440^a^		Covaci et al. (2001)
	Russia	58; 65	55; 58		3.0; 3.2	2.0; 3.0		180; 230	160; 190		0.5; 2.0	0.5; 1.0					240	200		Polder et al. (2008a)
	Russia	110; 130	93;110		4.5; 5.6	3.8; 5.7		740; 850	620; 660		0.4; 0.7	0.3; 0.7					750; 860	620; 660		Polder et al. (1998)
	Russia	100; 130			10; 18			800; 1000			0.45; 0.58						810; 1000			Tsydenova et al. (2007)
	Serbia				11; 38^a^	13; 56^a^					33; 50^a^	29; 38^a^					110; 190^a^	100; 130^a^		Vukavic et al. (2003)
	Slovak Republic	19–240	5.0–170					11–87	10–65		2.0–12	1.0–7.0					18–95	14–70		Veningerova et al. (2001)
	Slovak Republic	98; 102	79; 80					19; 20	15; 16								19; 20	15; 16		Yu et al. (2007)
	Spain	340^a^						8.3^a^												Pico et al. (1995)
	Spain		630; 910																	Ribas-Fito et al. (2005)
	Sweden	11.7	11.8					15	9.6								15	9.6		Aune et al. (2002)
	Sweden	8.2; 11	7.7					6.4; 32	6.3											Bergman et al. (2010)
	Sweden	15.1	13.7					15	10								15	9.7		Darnerud (2001)
	Sweden	9.4	8.8					7.9	7.2								7.9	7.2		Lignell et al. (2003)
	Sweden	15	14					14	12								14	12		Lignell et al. (2004)
	Sweden	31																		Lundén and Norén (1998)
	Sweden																			Norén et al. (1996)
	Sweden	6.3–8.5						3.5–6.4												Athanasiadou and Bergman (2008)
	Netherlands		100						80											Albers et al. (1996)
	Ukraine		150; 190						710; 750											Gladen et al. (1999)
	Ukraine		168						730									730		Gladen et al. (2003)
	UK	43	25					68	50								68	50		Harris et al. (1999)
	UK		18	17			0.2		17	15		0.6	0.8				16	18		Kalantzi et al. (2004)
	Western Europe and other States		2.3																	UNEP (2011)
The Americas	Antigua and Barbuda	5.3															5			UNEP (2009)
	Brazil	12			1			270			5						280			Paumgartten et al. (2000)
	Canada																			Dewailly et al. (1996)
	Canada			107																Dewailly et al. (2000)
	Canada	43	43		4.4	1.6		18	21		0.76	0.66					23	23		Newsome and Ryan (1999)
	Canada	15	8–12^a^		0.31			23	12–16^a^		1.0						24	19		Newsome et al. (1995)
	Canada and USA			12																Fitzgerald et al. (2001)
	Chile	11															6.4			UNEP (2009)
	Mexico					60–160			160–230									280–320		Elvia et al. (2000)
	Mexico																			Pardio et al. (1998)
	Mexico	92			310			610			380			240			750			Rodas-Ortiz et al. (2008)
	Mexico	53			19			430			84						530			Waliszewski et al. (1998)
	Mexico	25; 41		20; 35	1.0; 4.0			61; 90		44; 66	2; 4						63; 99		48; 74	Waliszewski et al. (1999a)
	Mexico	30		20				60		40							60		50	Sun et al. (2010)
	Nicaragua							6.0			1						7			Romero et al. (2000)
	Uruguay	14.															30			UNEP (2009)
	USA	15.		15																Greizerstein et al. (1999)
	USA	2.3		1.6	1.7	1.4		7.7	4.4		7.6	5.1		1.9			19	13		Johnson-Restrepo et al. (2007)
	USA			14																Fitzgerald et al. (2001)
	USA	9.6																		Kostyniak et al. (1999)
	USA	5.8; 6.6^a^	4.8; 5.6^a^					7.8; 14^a^	5.5; 11^a^											Weldon et al. (2011)

When concentrations from more than one sampling site in the same country and study have been reported, the concentrations are given as two values separated by a semicolon “;” (two concentrations) or a dash “–” (for more than two concentrations)

^a^Recalculated from fresh weight, assuming 4 % fat content

**Table 5:** Concentrations (ng/g fat) of oxychlordane, α-chlordane, γ-chlordane, and ∑chlordanes, in mothers’ milk, as reported in studies from around the world, 1995–2011, are presented

Region	Country	Oxychlordane		α-Chlordane		γ-Chlordane		∑Chlordanes		Reference
		Mean	Median	Mean	Median	Mean	Median	Mean	Median	
Africa	Africa								4.1	UNEP (2011)
	Ghana							1.2		UNEP (2009)
	Nigeria							2.4		UNEP (2009)
	Senegal							11.7		UNEP (2009)
Asia and the Pacific region	Asia and the Pacific								3.6	UNEP (2011)
	Australia	7.6	7							Khanjani and Sim (2006)
	Australia	2.8–18								Harden et al. (2007)
	Australia	5.1	5.1			5.5	5.4			Mueller et al. (2008)
	Australia	140; 150								Quinsey et al. (1995)
	Australia		7							Sim et al. (1998)
	China	0.49								Haraguchi et al. (2009)
	China	2.9; 8								Kunisue et al. (2004a)
	China		1.0; 1.0		1.2					Leng et al. (2009)
	Hong Kong and China	6.1						6.1		Hedley et al. (2010)
	India							2.6–3.4		Devanathan et al. (2009)
	Indonesia	0.49–2.4	0.49–0.73					7.0–30	5.5–8.3	Sudaryanto et al. (2006)
	Japan	3–4.8								Haraguchi et al. (2009)
	Japan							85		Konishi et al. (2001)
	Japan	14						58		Kunisue et al. (2006)
	Japan							0.76		Nagayama et al. (2007a)
	Jordan			460		590				Nasir et al. (1998)
	Korea	5.1								Haraguchi et al. (2009)
	Korea							3.7		UNEP (2009)
	Malaysia	7.1								Sudaryanto et al. (2005)
	Taiwan			10						Chao et al. (2006)
	Philippines	1.7–3.0	1.6–2.5	0.59–1.4	0.52–0.60					Malarvannan et al. (2009)
	Turkey	7^a^	5^a^							Erdogrul et al. (2004)
	Vietnam	0.047								Haraguchi et al. (2009)
	Vietnam	0.9; 2.4		0.8; 1.1		1.5; 3.7		2; 6.9		Minh et al. (2004)
	Vietnam		0.26; 0.51						0.4; 0.96	Nguyen et al. (2010)
Europe	Central and Eastern Europe								1.85	UNEP (2011)
	Denmark	5.1		0.03		0.05				Shen et al. (2007)
	Denmark		4.7		0.03		0.05			Shen et al. (2008)
	Finland	4.0		0.02		0.04				Shen et al. (2007)
	Finland		3.6		0.02		0.03			Shen et al. (2008)
	Finland and Denmark		4.3				0.050			Damgaard et al. (2006)
	Germany	5	4							Raab et al. (2008)
	Germany	8.8								Skopp et al. (2002)
	Norway	3.6–4.9	3.9–5.0	1.6–2.3	1.4–2.5					Polder et al. (2008b)
	Norway	3.0; 4.4	2.8; 3.2							Polder et al. (2009)
	Poland	3.3	2.8							Jaraczewska et al. (2006)
	Russia	5; 6	5; 5	1; 3	1; 2			21; 22	16; 20	Polder et al. (2008a)
	Russia	3.9; 5.6						10; 19		Tsydenova et al. (2007)
	Russia	7.9; 8.1		3.3; 6.9	3; 5.4			33; 59	21; 52	Polder et al. (1998)
	Sweden	2.5–3.5	2.4; 3.4							Athanasiadou and Bergman (2008)
	Sweden	4.4	3.6							Darnerud (2001)
	Sweden	3.3	3.1							Lignell et al. (2003)
	Sweden	4.4	3.8							Lignell et al. (2004)
	Ukraine		16; 22							Gladen et al. (1999)
	Ukraine		18							Gladen et al. (2003)
	UK				0.3					Kalantzi et al. (2004)
	Western Europe and other States								3.6	UNEP (2011)
The Americas	Antigua and Barbuda							4.4		UNEP (2009)
	Canada	59	43	1.3						Newsome and Ryan (1999)
	Canada	13	7.8–13^a^	0.21		0.16				Newsome et al. (1995)
	Chile							2		UNEP (2009)
	GROLAC^b^								4.4	UNEP (2011)
	Mexico		30–40							Elvia et al. (2000)
	Mexico			260		930		970		Rodas-Ortiz et al. (2008)
	Uruguay							4.4		UNEP (2009)
	USA	17	3.8	2.7	1.2	1.1				Johnson-Restrepo et al. (2007)

When concentrations from more than one sampling site in the same country and study have been reported, the concentrations are given as two values separated by a semicolon “;” (two concentrations) or a dash “–” (for more than two concentrations)

^a^Recalculated from fresh weight, assuming 4 % fat content

^b^Group of Latin America and Caribbean countries

**Table 6:** Concentrations (pg TEQs/g fat) PCDDs/PCDFs and DL-PCBs, in mothers’ milk, as reported in studies from around the world, 1995 – 2011 are presented^a^. Also the ∑TEQs levels are presented, giving data as reported in the studies referred to

Region	Country	∑PCDD/F				∑DL-PCB		Total-TEQ			
		mean		median		mean	mean	mean		median	
		(TEQ1998)	(TEQ2005)	(TEQ1998)	(TEQ2005)	(TEQ1998)	(TEQ2005)	(TEQ1998)	(TEQ2005)	(TEQ1998)	Reference
**Africa**											
	Africa				3.6						(UNEP 2011)
	Ghana									3.2	(UNEP 2009)
	Nigeria									3.1	(UNEP 2009)
	Senegal									7.2	(UNEP 2009)
**Asia and the Pacific region**											
	Asia & the Pacific				4.5						(UNEP 2011)
	China	15	9.0								(Chan et al. 2007)
	China							2.9-16	2.4-13		(Li et al. 2009)
	China	4.9	3.9			3.3	2.3	8.2	6.2		(Sun et al. 2011)
	China	4.4-6.2	1.9-3.9						6.2-7.5		(Sun et al. 2010)
	China	0.12	0.29								(Zheng et al. 2003)
	China			17; 17						23	(Sun et al. 2005)
	Hong Kong	8.2	7.2					13			(Hedley et al. 2006)
	Japan	13	11					21			(Kunisue et al. 2006)
	Japan							0.28			(Nagayama et al. 2007a)
	Japan	11	9.7					19			(Nakamura et al. 2008)
	Japan	19	10			6	7	25	18		(Saito et al. 2005)
	Japan	17	15			6.8	5.5	24	21		(Suzuki et al. 2005)
	Japan	15	13			11		26	13		(Tajimi et al. 2005)
	Japan	6.9	1.1			4.6	3.4	11	8.6		(Todaka et al. 2011)
	Japan									23	(Nagayama et al. 2007b)
	Kazakhstan	23; 46	21; 45								(Hooper et al. 1998)
	Korea								4.0		(UNEP 2009)
	Korea	10; 23	8.9; 21			4.8; 6.1	3.5; 4.8	15; 29	12; 25		(Yang et al. 2002)
	Taiwan	7.4; 12	0.73; 6.6					12			(Chao et al. 2005)
	Taiwan	15	13								(Hsu et al. 2007)
	Taiwan	7.6						13			(Wang et al. 2004)
	Turkey	4.9-12	3.9-10					6.8-16			(Cok et al. 2009)
	Uzbekistan			22							(Ataniyazova et al. 2001)
	Vietnam				2.7; 6.6						(Nhu et al. 2011)
	Vietnam	0.086; 0.13	0.073-0.12								(Tawara et al. 2011)
**Europe**											
	Belgium	29	16	32				41		42	(Focant et al. 2002)
	Central &										
	Eastern Europe				5.9						(UNEP 2011)
	Czech Rep.	13; 20	9.8; 15			6.0-9.8	4.7-8.6	19-30	15-23		(Bencko et al. 1998)
	Faroe Islands	36	31			14	14	50	46		(Grandjean et al. 1995)
	Finland			8.6; 12						14; 19	(Alaluusua et al. 2002)
	Finland	21; 26	19; 23			22; 29	13; 19	43; 55	32; 42		(Vartiainen et al. 1997)
	Germany	9.8	4.9					20	14		(Raab et al. 2008)
	Germany	14	11					27	11		(Wittsiepe et al. 2007)
	Italy	9.4-15	7.8-12					20-34			(Abballe et al. 2008)
	Italy	4.6-6.1	3.8-4.9			6.2-6.9	4.8-5.7	11-13	8.6-11		(Ulaszewska et al. 2011)
	Latvia	7.1-12	5.8-9.2			170; 190		170; 200			(Bake et al. 2007)
	Lithuania	14-18	12-15			30-30	16-17	44-48	28-31		(Becher et al. 1995)
	Norway	9.7-13	8.2-11			18-30	9.3-20	29-40	18-29		(Becher et al. 1995)
	Russia	16; 28	7.2; 9.5					27; 28			(Schecter et al. 2002)
	Slovak Rep.	5.7-12	4.5-9.0					14-24			(Chovancova et al. 2011)
	Spain							10.9			(Bordajandi et al. 2008)
	Spain				7.6		9		16.6		(Schuhmacher et al. 2009)
	Sweden	13	11			13	12	26	24		(Atuma et al. 1998)
	Sweden			9						13	(Darnerud et al. 2010)
	Sweden	8.8	7.6					19			(Glynn et al. 2001)
	Sweden	8.1	6.9	7.4	6.4	8.1	5.6	16	13	15	(Glynn et al. 2007)
	Sweden	8.2	7.0					16	13		(Lignell et al. 2009a)
	Sweden	21	15			20	13	40	28		(Lundén and Norén 1998)
	Sweden	3.5	2.9			3.6	2.2	7.0	5.0		(Fång et al. 2013)
	Western Europe & other States				6.0						(UNEP 2011)
**The Americas**											
	Antigua & Barbuda								4.3		(UNEP 2009)
	Brazil	10	10			4.4	0.60	14	11		(Paumgartten et al. 2000)
	Canada	4.9-14	3.3-8.3					6.5-18			(Newsome & Ryan 1999)
	Chile								9.7		(UNEP 2009)
	GROLAC^a^				5.6						(UNEP 2011)
	Uruguay								6.9		(UNEP 2009)
	**Country**	**∑PCDD/F**	**∑PCDD/F**	**∑PCDD/F**	**∑PCDD/F**		**Total-TEQ**		**Total-TEQ**		
		**(mean,TEQ1998)**	**(mean,TEQ2005)**	**(mean, I-TEQ)**	**(CALUX-TEQ)**		**(CALUX-TEQ)**		**(EROD-TEQ)**		
	Brazil^b^	10		8.1							(Paumgartten et al. 2000)
	China			9.4; 13	14; 15						(Leng et al. 2009)
	China^b^	0.12	0.29	1.0							(Zheng et al. 2003)
	Hong Kong								18		(Tsang et al. 2009)
	Germany			20; 23							(Schlaud et al. 1995)
	Kazakhstan^b^	23; 46		20; 40							(Hooper et al. 1998)
	Spain	12		10							(Schuhmacher et al. 2004)

^a^When concentrations from more than one sampling site in the same country and study have been reported the concentrations are given as two values separated by a semicolon “;” (two concentrations) or a hyphen “–“ (for more than two concentrations)

^b^Included for comparison with WHO TEQs, not unique samples

^c^Group of Latin America and Caribbean Countries

**Table 7:** Concentrations (ng/g fat) of BDE-47, BDE-209, and ∑PBDE, in mothers’ milk, as reported in studies from around the world, 1995–2011, are presented

Region	BDE-47				BDE-209		∑PBDE			Reference
	Country	Mean	Median	Geo. mean	Mean	Median	Mean	Median	Geo. mean	
Africa	Ghana	0.77–2.1	0.49–1.7		0.83–1.4	0.39–0.95	2.2–5.8	1.3–4.3		Asante et al. (2011)
	South Africa	0.29	0.3				1.7	1.4		Darnerud et al. (2011)
Asia and the Pacific region	China	0.89					1.9			Haraguchi et al. (2009)
	China	0.33; 0.66	0.32; 0.62		0.95; 1.3	1.8	4.7; 7.7	2.8; 7.1		Sudaryanto et al. (2008b)
	China	0.23–0.74	0.22–0.71		0.22–0.70	0.22–0.57	3.4–4.2	2.2–4.1		Sun et al. (2010)
	China	0.21–0.73					0.85–3.0			Zhang et al. (2011)
	Hong Kong and South China	1.9					3.4			Hedley et al. (2010)
	Indonesia	0.22–0.58			0.53; 0.54		0.91–1.8			Sudaryanto et al. (2008a)
	Japan	0.37					1.4			Akutsu et al. (2003)
	Japan	19	0.58				31	1.5		Akutsu and Hori (2004)
	Japan	0.57–0.76					1.3–1.7			Haraguchi et al. (2009)
	Japan	2.1^a^	0.43^a^				4.6	3		Kawashiro et al. (2008)
	Korea	2					3.7			Haraguchi et al. (2009)
	Korea	1.2					2.7			Kim et al. (2011b)
	Philippines	1.2; 4.9	1.1; 1.2		1.8	1.8	2.6; 10	2.7; 4.2		Malarvannan et al. (2009)
	Taiwan	0.58	0.52		0.48	0.36	3.5	3.3		Koh et al. (2010)
	Turkey	0.15^a^	0.1^a^				0.2^a^	0.1^a^		Erdogrul et al. (2004)
	Turkey	6.0	3.3				67	43		Ozcan et al. (2011)
	Vietnam	0.19					0.42			Haraguchi et al. (2009)
	Vietnam		0.13; 0.40			0.57; 2.3		1.1; 4.0		Tue et al. (2010)
Europe	Czech Republic	0.86	0.61							Kazda et al. (2004)
	France					1.5		2.7		Antignac et al. (2008)
	Germany	0.67	0.48				2.0	1.6		Raab et al. (2008)
	Italy	0.82					1.3			Alivernini et al. (2011)
	Norway	1.7	0.95		0.5	0.25	3.3	1.9		Eggesbo et al. (2011)
	Norway	1.7	1.3		0.22	0.13	3.8	3.2		Polder et al. (2008b)
	Norway	1.7				0.32	3.4	2.1		Thomsen et al. (2010)
	Poland	1.1	0.73				2.5	2		Jaraczewska et al. (2006)
	Russia	0.43; 0.65	0.36; 0.58		0.35	0.19	1.1; 1.2	0.96; 1.1		Polder et al. (2008a)
	Russia							0.96		Tsydenova et al. (2007)
	Spain						0.33			Bordajandi et al. (2008)
	Spain							2.5		Schuhmacher et al. (2009)
	Sweden	1.6; 2			0.2; 1.5		3.9; 4.8			Athanasiadou and Bergman (2008)
	Sweden	1.79	1.3				3.0	2.4		Aune et al. (2002)
	Sweden	0.93; 2.4	0.71		0.66		2.1; 4.3	1.9		Bergman et al. (2010)
	Sweden	1.9	1.7				3.2	3.2		Darnerud (2001)
	Sweden	0.92					2.4			Fängström et al. (2008)
	Sweden		1.2–1.8					2.2–3.3		Glynn et al. (2011)
	Sweden		1.2							Guvenius et al. (2003)
	Sweden	1.8	1.3				3.4	2.8		Lignell et al. (2003)
	Sweden	1.2	0.76							Lignell et al. (2009b)
	Sweden	1.9	1.5				3.5	2.9		Lignell et al. (2009a)
	Sweden	1.5	1.5				2.6	2.5		Lind et al. (2003)
	Sweden	2.3					4.0			Meironyté et al. (1999)
	UK		2.7	3.0				6.3	6.6	Kalantzi et al. (2004)
The Americas	USA		28					51		Daniels et al. (2010)
	USA	41	7.7				75	20		Johnson-Restrepo et al. (2007)
	USA	73			3.7	1.4	130			Park et al. (2011)
	USA	41	18		0.92		74	34		Schecter et al. (2003)
	USA	36	17		1.4	0.10	66	30		Schecter et al. (2005)
	USA	36	24				76	40		Schecter et al. (2010)

When concentrations from more than one sampling site in the same country and study are reported, the concentrations are given as two values separated by a semicolon (two concentrations) or a dash (for more than two levels)

^a^Recalculated from fresh weight, assuming 4 % fat content

**Table 8:** Concentrations (ng/g fat) of *cis*-HCL-epoxide, HCL-epoxide, and heptachlor, in mothers’ milk, as reported in studies from around the world, 1995–2011, are presented

Region	Country	*cis*-HCL-epoxide	HCL-epoxide		Heptachlor		Reference
		Mean	Mean	Median	Mean	Median	
Africa	Africa					2.25	UNEP (2011)
	Ghana				0.9		UNEP (2009)
	Nigeria				0.9		UNEP (2009)
	Senegal				1.3		UNEP (2009)
Asia and the Pacific region	Asia and the Pacific					0.55	UNEP (2011)
	Australia		2.2–17	7.4			Harden et al. (2007)
	Australia		5.9	6			Mueller et al. (2008)
	Australia		53; 78				Quinsey et al. (1995)
	Australia			7			Sim et al. (1998)
	Australia		9.9	7			Khanjani and Sim (2006)
	China	0.7					Hedley et al. (2010)
	Iran		54				Cok et al. (1999)
	Japan		7.5				Konishi et al. (2001)
	Japan		7.4				Saito et al. (2005)
	Japan		3				Nagayama et al. (2007a)
	Jordan		190		500		Nasir et al. (1998)
	Korea				2.2		UNEP (2009)
	Kuwait		1.3				Saeed et al. (2000)
	Taiwan		4.3		3		Chao et al. (2006)
	Thailand		160^a^	110^a^	110^a^	110^a^	Stuetz et al. (2001)
	Turkey		61				Cok et al. (2005)
	Turkey		38				Cok et al. (2011)
Europe	Central and Eastern Europe					0.50	UNEP (2011)
	Croatia		0.7				Frkovic et al. (1996)
	Denmark			2.9			Shen et al. (2008)
	Denmark	2.8					Shen et al. (2007)
	Denmark and Finland			2.3			Damgaard et al. (2006)
	Finland			2.0			Shen et al. (2008)
	Finland	2.2					Shen et al. (2007)
	Germany		4	3			Raab et al. (2008)
	Germany				21; 22		Schlaud et al. (1995)
	Germany		0.11				Zietz et al. (2008)
	Germany	7.3					Skopp et al. (2002)
	Netherlands			30			Albers et al. (1996)
	Spain				9^a^		Pico et al. (1995)
	Ukraine			22			Gladen et al. (1999)
	Ukraine			16			Gladen et al. (2003)
The Americas	Antigua and Barbuda				1.4		UNEP (2009)
	Brazil	8					Paumgartten et al. (2000)
	Canada		0.94				Newsome and Ryan (1999)
	Canada		3.8	0.75–3.2^a^			Newsome et al. (1995)
	Chile				1.7		UNEP (2009)
	GROLAC^b^					1.4	UNEP (2011)
	Mexico			40			Elvia et al. (2000)
	Mexico		160		580		Rodas-Ortiz et al. (2008)
	Nicaragua		6		1		Romero et al. (2000)
	Uruguay				1		UNEP (2009)
	Western Europe and other States					0.8	UNEP (2011)

When concentrations from more than one sampling site in the same country and study have been reported, the concentrations are given as two values separated by a semicolon “;” (two concentrations) or a dash “–” (for more than two concentrations)

^a^Recalculated from fresh weight, assuming 4 % fat content

^b^Group of Latin America and Caribbean countries

**Table 9:** Concentrations (ng/g fat) of aldrin, dieldrin, endrin, and ∑drins, in mothers’ milk, as reported in studies from around the world, 1995–2011, are presented

Region	Country	Aldrin		Dieldrin			Endrin		∑drins	Reference
		Mean	Median	Mean	Median	Geo. mean	Mean	Median	Mean	
Africa	Africa				2.8					UNEP (2011)
	Ghana			120						Ntow et al. (2008)
	Ghana			1.3						UNEP (2009)
	Nigeria			4.1						UNEP (2009)
	Senegal			3.1						UNEP (2009)
	Tunisia			25						Ennaceur et al. (2008)
	Tunisia			59	36					Ennaceur et al. (2007)
Asia and the Pacific region	Asia and the Pacific				1.8					UNEP (2011)
	Australia			51	40					Khanjani and Sim (2006)
	Australia	0.01–0.68		15						Harden et al. (2007)
	Australia	0.19	0.05	16	14					Mueller et al. (2008)
	Australia				25^a^					Noakes et al. (2006)
	Australia			150; 160						Quinsey et al. (1995)
	Australia				39					Sim et al. (1998)
	China			9300–10,000^a^						Wang et al. (2008)
	China								7.9	Zhou et al. (2011)
	China and Hong Kong			1.1						Hedley et al. (2010)
	India	250^a^								Siddiqui et al. (2002)
	Japan			28						Konishi et al. (2001)
	Japan				3					Nagayama et al. (2007a)
	Jordan	860		1400				3300		Nasir et al. (1998)
	Korea			1.3						UNEP (2009)
	Kuwait	5.2		4.2			4.0			Saeed et al. (2000)
Europe	Central and Eastern Europe				1.6					UNEP (2011)
	Croatia	1.3		1.0			2.0	0.7		Frkovic et al. (1996)
	Denmark					5.1				Shen et al. (2007)
	Denmark				4.9					Shen et al. (2008)
	Finland					2.8				Shen et al. (2007)
	Finland				2.4					Shen et al. (2008)
	Finland and Denmark				3.6					Damgaard et al. (2006)
	Germany			14						Schlaud et al. (1995)
	Germany			0.018–3.8	4					Zietz et al. (2008)
	Germany			4	2					Raab et al. (2008)
	Great Britain			48	25					Harris et al. (1999)
	Netherlands				50					Albers et al. (1996)
	Western Europe and other States				2.5					UNEP (2011)
The Americas	Antigua and Barbuda			2.6						UNEP (2009)
	Brazil			23						Paumgartten et al. (2000)
	Canada			11	1.1					Newsome and Ryan (1999)
	Canada			9.8	8.5					Newsome et al. (1995)
	Canada					30				Dewailly et al. (2000)
	Chile			5.0						UNEP (2009)
	Group of Latin America and Caribbean countries				4.9					UNEP (2011)
	Mexico				30–50					Elvia et al. (2000)
	Mexico	280		300			290			Rodas-Ortiz et al. (2008)
	Nicaragua			18			3.0			Romero et al. (2000)
	Uruguay			4.9						UNEP (2009)

When concentrations from more than one sampling site in the same country and study have been reported, the concentrations are given as two values separated by a semicolon “;” (two concentrations) or a dash “–” (for more than two concentrations)

^a^Recalculated from fresh weight, assuming 4 % fat content

**Table 10:** Concentrations (ng/g fat) of pentachlorobenzene (PCBz), toxaphene, and mirex, in mothers’ milk, as reported in studies from around the world, 1995–2011, are presented

Region	Country	PCBz			∑Toxaphene		Mirex			Reference
		Mean	Median	Geo. mean	Mean	Median	Mean	Median	Geo. mean	
Africa	Nigeria				4.1					UNEP (2009)
Asia and the Pacific region	Australia							0.21		Harden et al. (2007)
	Australia							0.18		Mueller et al. (2008)
	China							2.4		Zhou et al. (2011)
	Hong Kong and South China				0.8					Hedley et al. (2010)
	Korea					0.8				UNEP (2009)
Europe	Denmark			0.36			0.23			Shen et al. (2007)
	Denmark		0.32					0.21		Shen et al. (2008)
	Finland			0.27			0.31			Shen et al. (2007)
	Finland		0.25					0.26		Shen et al. (2008)
	Finland and Denmark		0.28					0.22		Damgaard et al. (2006)
	Germany				16					Skopp et al. (2002)
	Norway									Polder et al. (2008b)
	Russia				10; 20	10; 19	0.5; 0.8	0.5; 0.7		Polder et al. (2008a)
The Americas	Antigua and Barbuda				1.3					UNEP (2009)
	Canada								14	Dewailly et al. (2000)
	Canada								3.0	Fitzgerald et al. (2001)
	Canada	1.0	0.91		68	56	2.3	1.8		Newsome and Ryan (1999)
	Canada	1.5^a^	1.2^a^				1.9^a^	1.6^a^		Newsome et al. (1995)
	Mexico						200			Rodas-Ortiz et al. (2008)
	Uruguay						9.8			UNEP (2009)
	USA							1.0–5.8		Madden and Makarewicz (1996)
	USA								1.4	Fitzgerald et al. (2001)
	USA							2.4		Greizerstein et al. (1999)
	USA						4.8			Kostyniak et al. (1999)

When concentrations from more than one sampling site in the same country and study have been reported, the concentrations are given as two values separated by a semicolon “;” (two concentrations)

^a^Recalculated from fresh weight, assuming 4 % fat content

**Table 11:** Concentrations (ng/g fat) of HBCDD and PBB, in mothers’ milk, as reported in studies from around the world, 1995–2011, are presented

Region	Country	α-HBCDD		β-HBCDD		γ-HBCDD		∑HBCDD		∑PBB	Reference
		Mean	Median	Mean	Median	Mean	Median	Mean	Median	Mean	
Africa	Ghana	0.29–0.79	0.23–0.62	0.010				0.30–0.80	0.27–0.62		Asante et al. (2011)
	South Africa							0.55	0.34		Darnerud et al. (2011)
Asia	China	0.33–2.8				0.46		0.33–2.8			Shi et al. (2009)
	Japan	1.4						1.4			Kakimoto et al. (2008)
	Philippines	0.58; 0.72	0.50; 0.67	0.052; 0.18	0.043; 0.12	0.14; 0.48	0.13; 0.23	0.81; 1.0	0.52; 0.89		Malarvannan et al. (2009)
	Vietnam		0.33; 0.38						0.33; 0.38		Tue et al. (2010)
Europe	Denmark									0.26	Shen et al. (2008)
	Finland									0.17	Shen et al. (2008)
	Norway							1.7	0.86		Thomsen et al. (2010)
	Russia							0.47; 0.71	0.45; 062		Polder et al. (2008a)
	Spain	14	4.4			40	23	47	27		Eljarrat et al. (2009)
	Sweden							0.45	0.30		Aune et al. (2002)
	Sweden							0.63^a^; 0.80^a^	0.58^a^		Bergman et al. (2010)
	Sweden							0.39			Fängström et al. (2008)
	Sweden								0.3–0.4		Glynn et al. (2011)
	Sweden							0.42	0.35		Lignell et al. (2003)
	UK	4.9	3.2	0.32	0.30	0.49	0.50	6.0	3.8		Abdallah and Harrad (2011)

When concentrations from more than one sampling site in the same country and study have been reported, the concentrations are given as two values separated by a semicolon “;” (two concentrations) or a dash “–” (for more than two concentrations)

^a^Quantified using BDE-139 as surrogate standard

**Table 12:** PFOS concentrations in mothers’ milk, expressed in picograms per milliliter of milk as reported in studies from around the world, 1995–2011, are presented

Region	Country	Mean (pg/mL)	Median (pg/mL)	Reference
Asia and the Pacific	Cambodia	67	40	Tao et al. (2008a)
	China	6–140		Liu et al. (2010)
	China	120	100	So et al. (2006)
	India	46	39	Tao et al. (2008a)
	Indonesia	84	67	Tao et al. (2008a)
	Japan	230	200	Tao et al. (2008a)
	Malaysia	120	110	Tao et al. (2008a)
	Philippines	98	100	Tao et al. (2008a)
	South Korea	61		Kim et al. (2011a)
	Vietnam	76	59	Tao et al. (2008a)
Europe	Germany	120; 130	110; 120	Volkel et al. (2008)
	Germany	40		Fromme et al. (2010)
	Hungary	310	330	Volkel et al. (2008)
	Spain	120	110	Kärrman et al. (2010)
	Spain	120		Llorca et al. (2010)
	Sweden	120–260	170	Kärrman et al. (2007)
	Sweden	120		Kärrman et al. (2006)
	Sweden	75		Sundström et al. (2011)
The Americas	USA	130	110	Tao et al. (2008a)

When concentrations from more than one sampling site in the same country and study have been reported, the concentrations are given as two values separated by a semicolon “;” (two concentrations) or a dash “–” (for more than two concentrations)

**Table 13:** Concentrations (ng/g fat) of endosulfan, in mothers’ milk, as reported in studies from around the world, 1995–2011, are presented

Region	Country	α-Endosulfan		β-Endosulfan		∑Endosulfan		Endosulfan sulfate		Reference
		Mean	Median	Mean	Median	Mean	Median	Mean	Median	
Africa	Egypt		4.8							Saleh et al. (1996)
Asia	India					9100^a, b^				Sanghi et al. (2003)
	Turkey	50^a^		950^a^		1000^a^				Cok et al. (2011)
Europe	Spain	17^a^	22^a^	270^a^	180^a^	280^a^	200^a^	150^a^	120^a^	Cerrillo et al. (2005)
	Denmark	7.4								Shen et al. (2007)
	Denmark		7.4							Shen et al. (2008)
	Denmark and Finland		6.8							Damgaard et al. (2006)
	Finland	7.3								Shen et al. (2007)
	Finland		6.4							Shen et al. (2008)
The Americas	Mexico			280						Rodas-Ortiz et al. (2008)

When concentrations from more than one sampling site in the same country and study have been reported, the concentrations are given as two values separated by a semicolon “;” (two concentrations) or a dash “–” (for more than two concentrations)

^a^Recalculated from fresh weight, assuming 4 % fat content

^b^Nonspecified isomer/s

- One reported concentration from one sample location is represented by a single value, e.g., “5.”
- Two reported concentrations from one location are represented by two values separated by a semicolon, e.g., “3; 5.”
- Three or more reported concentrations from one sample location are represented by giving the range, e.g., “3–5.”
- If a study reports data from more than one sampling location, all are included, e.g., by presenting, “Sweden 5” and “Norway 4.”
- In the case of a time series, i.e., more than one sample from one location, only the most recent value is included in the summary table.
- A “sum value” is only given if more than one of the components of the “sum value” are reported.
- If “sum values” are reported, the reported value is used. If not reported, the sum is calculated if possible.
- If data from the same samples are presented in several studies, only the latest study is included in the table.
- Three-letter country codes according to ISO 3166-1 alpha-3 are used in the figures, herein.

### Spatial distribution diagrams

If mean or median values are given for different sampling locations within the same country or subgroups (e.g., age, primiparae versus multiparae), a weighted mean or median value is calculated based on the number of individuals in each group: for example, reported mean concentrations of 2, 3, and 5 ng/g fat of BDE-47, based on 10, 10, and 20 samples (total of 40 samples) from cities X, Y and Z in Sweden, respectively. The weighted mean value for study A will thus be a bar at 3.75 ng/g fat example given below.

$$ \frac{2\mathrm{ng}/\mathrm{g}\times 10}{40}+\frac{3\mathrm{ng}/\mathrm{g}\times 10}{40}+\frac{5\mathrm{ng}/\mathrm{g}\times 20}{40}=3.75\mathrm{ng}/\mathrm{g} $$

Equation 1. Example of how the weighted means were calculated.

In the spatial distribution diagrams, e.g., Fig. 3, the studies are sorted by rising concentrations within each region.

### Methods applied for statistical reports

To test for significant log-linear trends, log-linear regression analyses were performed for the entire investigated time period and for the most recent 10 years using the annual arithmetic mean values. In cases where the regression line had a poor fit, a 3-point running mean smoother was checked for statistical significance in comparison with the regression through an ANOVA (Nicholson et al. 1998). Potential outliers in the temporal trends were detected using a method described by Hoaglin and Welsch (1978). The suspected outliers are merely indicated in the figures and were included in the statistical calculations. Values below level of quantification (LOQ) were replaced by LOQ/2 prior to the statistical analyses. Power analysis was also carried out. The power was fixed to 80 % and the minimum possible trend to be detected during a monitoring period of 10 years at a significant level of 5 % was estimated. A significance level of 5 % was used for all tests.

## Results and discussion

**Fig. 1:**
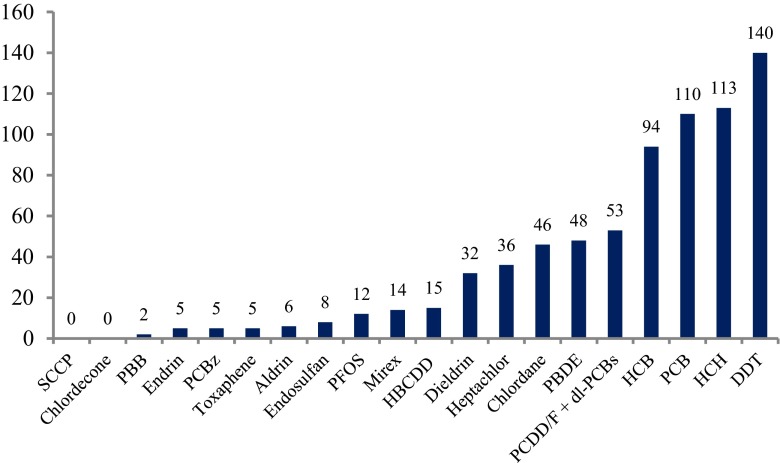
Number of reported observations (total 744) in 253 scientific papers of the legacy POPs in mothers’ milk, from 1995 to 2011, subdivided on the POPs reported herein and presented in order of abundance of studies. Eighty percent of all studies are linked to seven of the POPs, DDT—chlordane. Note that a scientific paper may include observations of more than POP

A total of 253 scientific articles on POPs in mothers’ milk were identified on the basis of the applied methodology (cf. above). Several of the articles included data on more than one of the POPs. The diagram (Fig. 1) visualizes the number of reported concentrations of the corresponding POP that were available for this review. The results are presented in this review in descending order, starting from the POPs that are most well researched in relation to occurrence and concentrations in mothers’ milk, worldwide, i.e., going from DDT to SCCP and chlordecone (Fig. 1).

**Fig. 2:**
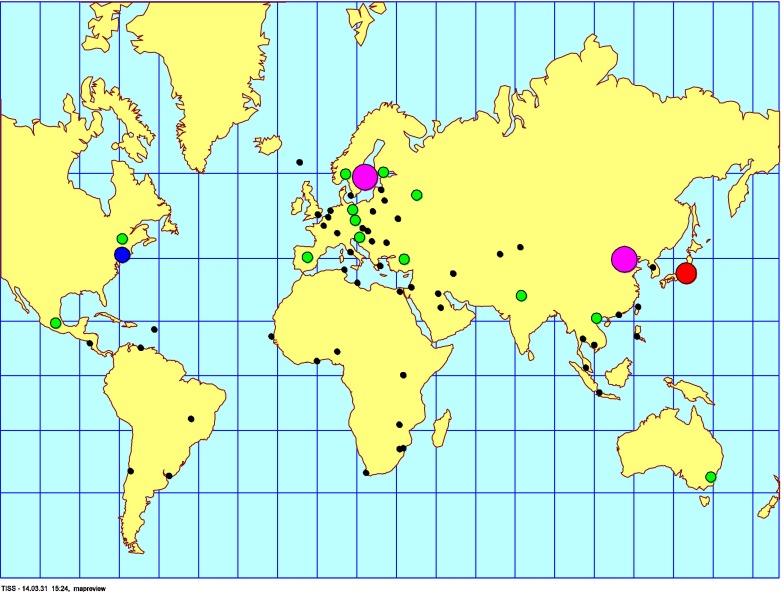
Global distribution of exposure assessment studies of POPs in mothers’ milk, up to year 2011. The *circles* are placed at the site of the capital city for each country, and the sizes of the *circles* visualize the abundance of studies from the countries on which this review is built. *Black circle* = 1 study, *green circle* = 2–5 studies, *blue circle* = 6–10 studies, *red circle* = 11–15 studies, and *pink circle* >15 studies

Looking into the distribution of the scientific articles published on POPs in mothers’ milk in the chosen time period, it is clear that most of the studies originate from China, Japan, North America, and Western Europe (Fig. 2). However, publications are scattered throughout the globe making a spatial trend review possible. The results of the spatial distribution and concentrations of POPs are presented under the sections “DDT and DDT-related compounds” to “SCCPs,” including tables and figures when applicable. Temporal trend data on POPs in mothers’ milk are scarce but available data are reported herein under the sections “DDT and DDT related compounds” to “PFOS.” Some novel data are included for recent exposure assessments performed on mothers’ milk from Sweden.

All concentration data given in Tables 2, 3, 4, 5, 6, 7, 8, 9, 10, 11, 12, and 13 are presented on a weight basis (ng or pg) per gram extracted fat, with the exception of perfluorooctane sulfonate (PFOS) which is presented in pg/mL.

It is of importance to consider that a reported concentration might not be generally applicable to a country as a whole, for instance, samples might originate from a farming area where pesticides have been in use. This could be more important to consider for countries with large diversity, either geographically and/or cultural, i.e., rural versus urban life styles. In smaller, more homogenous countries such as Sweden, POP concentrations have been found to be quite uniform, independent of geographical distribution (Glynn et al. 2011).

### DDT and DDT-related compounds

A very large total number of reports are dealing with DDT and related compounds in mothers’ milk. Related compounds are 2,4′-DDT and the transformation products 4,4′-DDE, 4,4′-DDD, 2,4′-DDE, and 2,4′-DDD. The data shown in Table 2 refer only to the three individual compounds 4,4′-DDT, 4,4′-DDE, and 4,4′-DDD as well as ∑DDT. However, the ∑DDT may consist of some very different sums, sometimes including only the three main 4,4′-substituted DDTs mentioned but occasionally also including 2,4′-substituted DDTs. This makes the sum data less reliable for comparisons. However, we have still chosen to include sum data to visualize the larger data set, but avoiding the confusion with further differentiated data. Concentration data on 4,4′-DDT, 4,4′-DDE, and 4,4′-DDD are presented in detail in Table 2, subsectioned into four geographically large areas, i.e., Africa; Asia, Australia, and the Pacific region; Europe; and The Americas. Some of the results on DDT and related compounds in mothers’ milk are highlighted below.

**Fig. 3:**
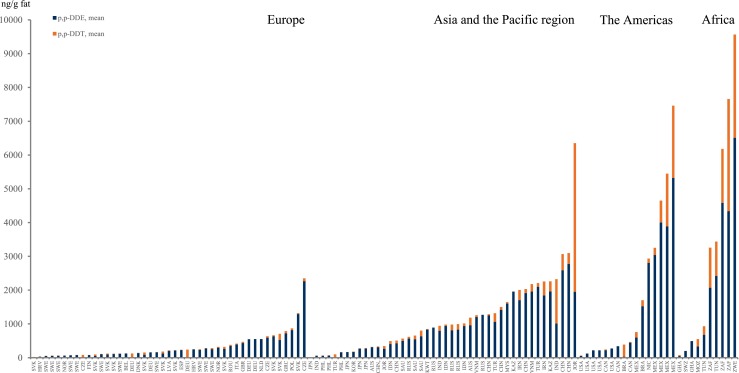
The sum of p,p-DDE and p,p-DDT reported worldwide is given in the figure, where contribution p,p-DDE is represented in *dark blue* and the contribution p,p-DDT is represented in *orange*

Table 2 includes calculated ratio values of 4,4′-DDT/4,4′-DDE based on reported mean or median concentrations of the two compounds and gives an indication for recent discharges of DDT (with ratio values of 0.5 and above) or more historical use (ratios below 0.2) of this pesticide. Figure 3 displays the 4,4′-DDT and 4,4′-DDE concentrations as reported throughout the world.

#### Africa

Concentrations of 4,4′-DDE are the highest among the three individual DDT compounds reported in Table 2, although the 4,4′-DDT/4,4′-DDE ratio indicates similar levels between the two major constituents in mothers’ milk. The highest concentrations of 4,4′-DDT and 4,4′-DDE are reported from Zimbabwe (Chikuni et al. 1997) and South Africa (Okonkwo et al. 2008; Sereda et al. 2009). Still, a few studies indicate low concentrations of DDTs in mothers’ milk. It is particularly clear that African mothers have high concentrations of 4,4′-DDT compared to most other samples from other regions. This is of course implying present or recent use of DDT for spraying, potentially indoors (Channa et al. 2012).

#### Asia, Australia, and the Pacific region

Reports from Asia, Australia, and the Pacific Region indicate that certain mothers have been highly exposed to both 4,4′-DDT and 4,4′-DDE (Nair et al. 1996; Nasir et al. 1998; Stuetz et al. 2001; Wong et al. 2002), while overall levels are above the common European concentrations, but below the concentrations reported for milk from African mothers (Table 2 and Fig. 3). The data indicate primarily old releases of DDT based on the low 4,4′-DDT/4,4′-DDE ratio, although there are exceptions (e.g., Nair et al. 1996; Nasir et al. 1998; Stuetz et al. 2001; Wong et al. 2002). Accordingly, direct exposure to DDT cannot be excluded.

#### Europe

The majority of studies on DDT in mothers’ milk are originating from Europe. The levels are in the lowest end (e.g., 20–250 ng/g fat of 4,4′-DDE) of all studies reviewed except for studies on DDTs in milk from the Eastern part of Europe, 250–2800 ng/g fat, as shown in Table 2. The higher concentrations in Eastern Europe is also followed by higher 4,4′-DDT/4,4′-DDE ratios indicating more recent use or unintentional release of DDT. However, the ratio is generally low indicating successful elimination of this POP from use in the society. The DDT and related compounds still present in mothers’ milk are a mirror of intake via food. Some high exposure levels to DDT among Eastern European citizens, as determined by analysis of blood, are supporting the higher levels in mothers’ milk from countries in this part of Europe (Hovander et al. 2006).

#### The Americas

Low concentrations of DDTs are reported from Canada and the USA, while Mexico in Central America (Table 2) reported levels that are similarly high as in Africa and some Asian countries. It is notable that in the countries from which the mothers’ milk contain the highest concentrations of DDTs, there is a more recent input of DDT (Fig. 3), which is confirmed by higher 4,4′-DDT/4,4′-DDE ratios, 0.12–0.4 (Table 2). A Brazilian study is reporting the highest ratio among all studies reviewed, i.e., 12 (Azeredo et al. 2008), indicating the present use of DDT. However, the actual concentration of ∑DDTs is lower than many other studies.

### PCBs

**Fig. 4:**
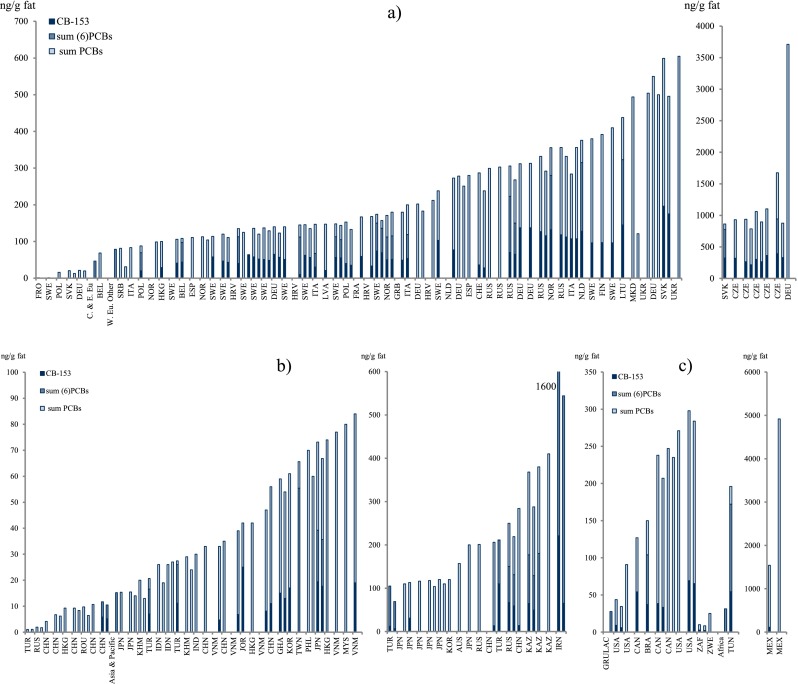
**a**–**c** Graphical presentation of PCB concentrations in mothers’ milk from countries worldwide

Polychlorinated biphenyls (PCBs) reported as CB-153, sum of the six indicator CBs (CB-28, CB-52, CB-101, CB-138, CB-153, and CB-180, only if the concentrations of all six were reported), or the estimated total sum of PCB (∑PCB, the method of estimating the sum may vary between studies) in all 116 studies were tabulated (Table 3). Dioxin-like PCBs are not reported here but instead discussed together with the dioxins and furans (“PCDDs, PCDFs, and DL-PCBs”). Since PCBs are showing decreasing trends after the bans came into effect, studies from different time periods (1995–2011) may not be altogether comparable (Fig. 4).

#### Africa

Five studies of PCBs from Africa were found in the database search. CB-153 ranges from approximately 2 to 120 ng/g fat in South Africa (Darnerud et al. 2011) and Tunisia (Ennaceur et al. 2008), respectively. The reported ∑PCB ranges from about 3 to 750 ng/g fat in Zimbabwe (Chikuni et al. 1997) and Tunisia (Ennaceur et al. 2008), respectively.

#### Asia, Australia, and the Pacific region

No less than 36 studies were found from this region and the majority report estimated ∑PCB. The lowest concentrations are from China (Kunisue et al. 2004a; Xing et al. 2009), India (Devanathan et al. 2009), Indonesia (Sudaryanto et al. 2006), and Cambodia (Kunisue et al. 2004a). Higher concentrations of ∑PCB were reported from Australia, 160–480 ng/g fat (Quinsey et al. 1995); Japan, 120–200 ng/g fat (Kawashiro et al. 2008; Kunisue et al. 2006; Nagayama et al. 2007a; Nakamura et al. 2008); Kazakhstan, 220–820 ng/g fat (Hooper et al. 1997; Lutter et al. 1998; She et al. 1998); and Russia, 160–240 ng/g fat (Tsydenova et al. 2007). The lowest concentrations of CB-153, 0.5 ng/g fat, were reported from China (Zhang et al. 2011) and the highest in Iran, over 200 ng/g fat (Behrooz et al. 2009).

#### Europe

Over 50 % of the included studies came from Europe. The lowest concentrations of CB-153 in Europe, lower than 50 ng/g fat, came from Belgium (Colles et al. 2008), Italy (Ulaszewska et al. 2011), Latvia (Bake et al. 2007), Norway (Polder et al. 2008b), Poland (Jaraczewska et al. 2006), and Sweden (Lignell et al. 2003). The highest concentrations (more than 300 ng/g fat) were reported from the Czech Republic (Bencko et al. 1998; Cerna et al. 2010; Schoula et al. 1996) and Slovak Republic (Petrik et al. 2001). This is also true for the estimated ∑PCB, when reported. Concentrations >1000 ng/g fat are reported from the Czech Republic (Bencko et al. 1998; Cerna et al. 2010; Schoula et al. 1996), Germany (Schlaud et al. 1995), and the Slovak Republic (Petrik et al. 2001). Although the ban of PCB that was introduced stepwise during the 1970s and 1980s has led to significantly lowered concentrations in the environment, leakage due to inappropriate handling of waste material or from, e.g., building material, large capacitors, and hydraulic systems, still in use or stored at dumping sites, can still be expected and can thus cause elevated concentrations in mothers’ milk from highly industrialized countries.

#### The Americas

The concentrations of CB-153 reported from most of the 14 studies from the Americas were fairly low to moderate, around or below 50 ng/g fat: Brazil (Paumgartten et al. 2000), Canada (Dewailly et al. 1996; Newsome et al. 1995), and the USA (Fitzgerald et al. 1998; Pan et al. 2010). The highest concentration of CB-153, 110 ng/g fat (Rodas-Ortiz et al. 2008), as well as of the estimated ∑PCB, 1500 ng/g fat (Rodas-Ortiz et al. 2008), was reported from Mexico.

### HCB and HCHs

Mothers’ milk concentrations of hexachlorobenzene (HCB) and the three more common hexachlorocyclohexane (HCH) isomers, α-HCH, β-HCH, and γ-HCH, are presented in Table 4, as well as the less commonly reported levels of δ-HCH. α-HCH, β-HCH, and γ-HCH represent the HCHs present in the “old” technical-grade HCH pesticide, while the commonly used pesticide, lindane, corresponds to γ-HCH. All the HCH isomers are related to pesticide use, while HCB has both a pesticide history and is also an abundant by-product from industrial activities and poorly controlled incineration/backyard burning. The pattern of HCH in the world is highly influenced by recent use of HCH as a pesticide. It is notable that the β-HCH isomer is the most abundant of the HCH isomers in mothers’ milk even though this compound is related to the historical HCH pesticide use and not to lindane (γ-HCH). However, the half-life of γ-HCH is much shorter in humans and wildlife than the half-life of β-HCH, and the observations confirm the higher persistency and lower reactivity of the β-HCH isomer compared to the others.

**Fig. 5:**
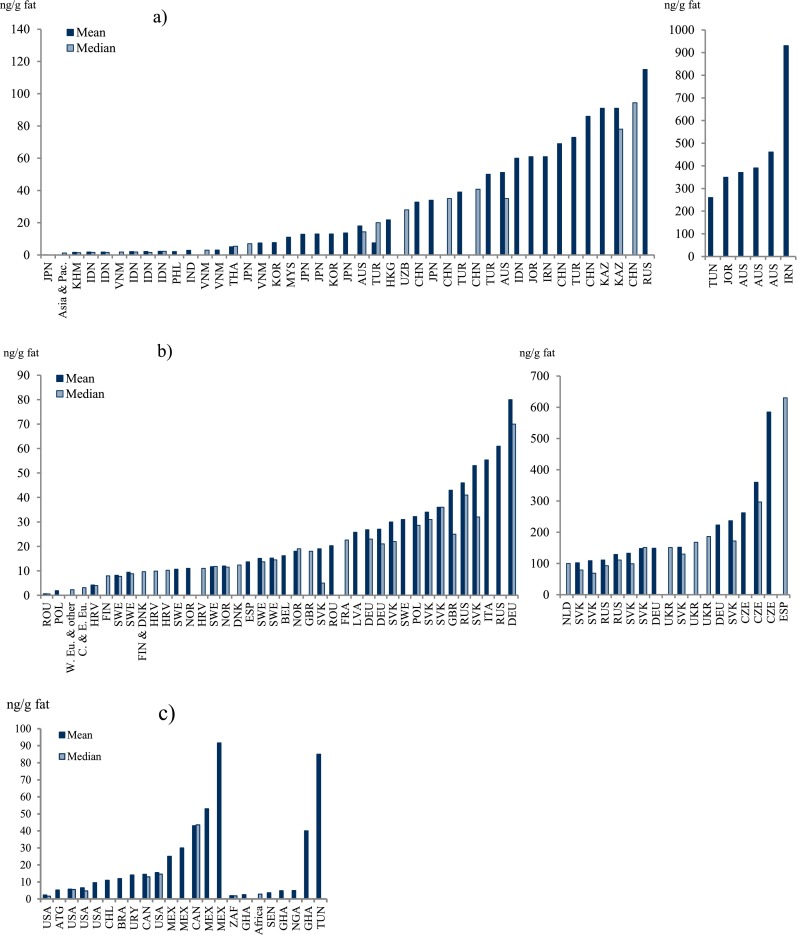
**a**–**c** Graphical presentation of HCB concentrations in mothers’ milk from countries worldwide

**Fig. 6:**
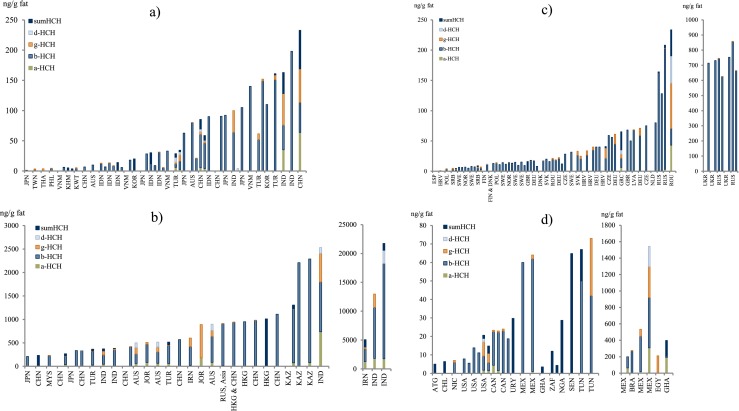
**a**–**d** Graphical presentation of HCH concentrations in mothers’ milk from countries worldwide. Note that “RUS, Asia” refers to samples from a location in the Asian part of the Russian Federation

The data for HCB and the HCHs are dominated by studies of mothers’ milk from Asia and Europe (Table 4) and are reported in 94 and 113 scientific reports worldwide, respectively (Figs. 5 and 6).

#### Africa

Most reported HCB concentrations from African countries range from about 2 to 5 ng/g fat, on either mean or median basis. Somewhat higher levels are reported in Ghananese mothers’ milk, 2.5–40 ng/g fat (Darko and Acquaah 2008; Ntow 2001; UNEP 2009). The highest concentrations are reported from Tunisia, mean concentration of 0.4–290 ng/g fat (Ennaceur et al. 2007, 2008).

In general, one could consider the ∑HCH levels in African mothers’ milk to be on the lower end on a global scale and comparable to concentrations reported from the Americas. There are quite few studies from Africa reporting levels of ∑HCH in mothers’ milk. Most of the studies report mean concentrations of approximately 10–100 ng/g fat (Table 4), the exceptions being one study from Ghana (Saleh et al. 1996) and one study from Libya (Elafi et al. 2001), reporting concentrations of 210 and 500 ng/g fat, respectively.

#### Asia, Australia, and the Pacific region

A few countries have reported HCB mean concentrations below 10 ng/g fat: Cambodia (Kunisue et al. 2004a), India (Devanathan et al. 2009), Korea (UNEP 2009), and the Philippines (Malarvannan et al. 2009). However, the majority of countries have reported mean values in the range 10–100 ng/g fat (Table 4). In the higher end of reported HCB, concentrations from the region include Australian levels between 370 and 460 ng/g fat (Behrooz et al. 2009; Quinsey et al. 1995), with levels up to 1500 ng/g fat, as well as Kazakhstan (Hooper et al. 1997) and Thailand (Stuetz et al. 2001), reporting concentrations above 100 ng/g fat (Table 4).

The reported levels of HCHs, both individual isomers as well as ∑HCH, are the highest in Asia, Australia, and the Pacific region, compared to the rest of the world, although there are a few studies reporting comparatively low mean concentrations, i.e., below 10 ng/g fat: Cambodia (Kunisue et al. 2004a), Kuwait (Saeed et al. 2000), the Philippines (Malarvannan et al. 2009), and Taiwan (Chao et al. 2006) but also studies from Japan (Nagayama et al. 2007a) and Australia (Khanjani and Sim 2006; Kunisue et al. 2004a). On the contrary, other studies from Japan and Australia report higher concentrations (Table 4). The majority of studies from Asia, Australia, and the Pacific region report ∑HCH concentrations in the range of hundreds of nanogram per gram fat, but a large number report concentrations in the range of thousands of nanogram per gram fat (Table 4). In India, there is a high HCH contamination according to the mothers’ milk concentrations, which range from 120 to 22,000 ng/g fat ∑HCH, with several studies reporting values of thousands of nanogram per gram fat (Table 4). The highest concentrations of the HCH isomers in mothers’ milk have been reported from India with means of 1800, 16,000, 1300, and 2300 ng/g fat for α-HCH, β-HCH, γ-HCH, and δ-HCH, respectively (Siddiqui et al. 2002).

#### Europe

In general, the HCB concentration in European mothers’ milk is higher than the rest of the world, although the most extreme values of HCB in mothers’ milk are not from Europe. Only two European countries, Croatia (Frkovic et al. 1996) and Finland (Shen et al. 2007), report mean HCB concentrations below 10 ng/g fat, while several Croatian studies report median concentrations above 10 ng/g fat (Krauthacker et al. 1998, 2009; Romanic and Krauthacker 2006). The majority of the HCB concentrations reported from European countries are in the range of 10–100 ng/g fat, reported on either mean and median basis (Table 4). In Europe, it is primarily the Eastern countries that report highly elevated HCB levels in the analyzed mothers’ milk, with the highest levels from the Czech Republic, median values up to 370 ng/g fat (Cajka and Hajslova 2003; Cerna et al. 2010). However, high levels are also reported in Spanish mothers’ milk with medians of 630 and 910 ng/g fat (Ribas-Fito et al. 2005).

The HCH concentrations in mothers’ milk from European mothers in general show significantly lower concentrations than milk from Asia, Australia, and the Pacific regions, but higher than concentrations reported from Africa and the Americas. The reported European levels of ∑HCH are for the most part homogenous, and the majority of mean and/or median concentrations are in the range 10–100 ng/g fat. However, there are a number of studies from Denmark (Shen et al. 2007), Finland (Shen et al. 2007), Romania (Covaci et al. 2001), and Russia (Polder et al. 1998; Tsydenova et al. 2007) that report ∑HCH concentrations of several hundreds of nanogram per gram fat (Table 4). The highest reported mean concentration is 1000 ng/g fat, in a study from Russia (Tsydenova et al. 2007).

#### The Americas

Overall, the HCB contamination seems to be lower in the Americas than any of the other regions with a higher portion of studies below 10 ng/g fat and no study reporting concentrations above 100 ng/g fat (Table 4). Antigua and Barbuda (UNEP 2009) along with a number of studies from the USA report levels below 10 ng/g fat. However, two studies report a mean concentration of 15 ng/g fat (Greizerstein et al. 1999) and a median concentration of 14 ng/g fat (Fitzgerald et al. 2001). The two highest mean concentrations of HCB were reported in studies of Mexican mothers’ milk, reaching 92 and 53 ng/g fat (Rodas-Ortiz et al. 2008; Waliszewski et al. 1998), and the third highest was reported from Canada, 43 ng/g fat (Newsome and Ryan 1999).

In the Americas, the ∑HCH concentrations are similar to the concentrations in Europe, albeit there are fewer reported observations. The two lowest concentrations are from Antigua and Barbuda (UNEP 2009) and Nicaragua (Romero et al. 2000), 5 and 7 ng/g fat, respectively. The majority of studies report values in the lower end of the range 10–100 ng/g (Table 4), although exceptions to this are reported concentrations in the range of hundreds of nanogram per gram from Brazil (Paumgartten et al. 2000) and Mexico (Elvia et al. 2000; Rodas-Ortiz et al. 2008; Waliszewski et al. 1998), with the highest mean concentration reported in mothers’ milk from Mexico, at 750 ng/g fat.

### Chlordane

Chlordane concentrations reported as oxychlordane, α-chlordane, γ-chlordane, and ∑chlordanes from 63 studies, were selected and tabulated (Table 5).

#### Africa

Only ∑chlordanes from four countries on the African continent were reported. The highest concentrations were from Senegal, with a mean concentration of 11.7 ng/g fat (UNEP 2009).

#### Asia, Australia, and the Pacific region

Concentrations of oxychlordane vary greatly between countries, mostly between 0.5 and 10 ng/g fat. Extreme concentrations (140 and 150 ng/g fat) are reported from one Australian study (Quinsey et al. 1995), whereas the other studies from Australia report concentrations below 20 ng/g fat. Banned in most countries in 1997, chlordane was still allowed to be used as a termiticide in the Northern Territory (Australia) (UNEP Chemicals). Concentrations of α- and γ-chlordane are only reported from a few countries, whereof extreme concentrations are reported from Jordan 460 and 590 ng/g fat, respectively (Nasir et al. 1998). The highest concentrations of ∑chlordanes were reported from Japan (Konishi et al. 2001; Kunisue et al. 2006), while studies from the rest of the countries in the region report concentrations generally below 10 ng/g fat.

#### Europe

Fourteen studies from Europe report mean concentrations of oxychlordane, most of them close to or below 5 ng/g fat, and the reported median concentrations are in general of the same magnitude. The highest values of oxychlordane in Europe are reported from Ukraine (16–22 ng/g fat) (Gladen et al. 1999, 2003). Only very few countries reported ∑chlordanes, whereof the highest values, 10–60 ng/g fat, are reported from Russia (Polder et al. 1998, 2008a; Tsydenova et al. 2007). One study reports concentrations below 4 ng/g fat from Western, Central, and Eastern Europe (UNEP 2011).

#### The Americas

A few studies from the American continent in general show higher concentration of oxychlordane (Johnson-Restrepo et al. 2007; Newsome et al. 1995; Newsome and Ryan 1999) compared to Europe. The highest concentrations are between 40 and 60 ng/g fat from Canada (Newsome and Ryan 1999) and Mexico (Elvia et al. 2000). A few studies reporting concentrations of α- and γ-chlordane give values below 3 ng/g fat, except extreme concentrations reported from Mexico, 260 and 930 ng/g fat, respectively (Rodas-Ortiz et al. 2008).

### PCDDs, PCDFs, and DL-PCBs

The polychlorinated dibenzo-*p*-dioxin/polychlorinated dibenzofuran (PCDD/PCDF) and dioxin-like PCB (DL-PCB) concentrations are reported as toxic equivalents (TEQs) based on WHO TEF values from 1998 (Van den Berg et al. 1998) and 2005 (Van den Berg et al. 2006). Total mean TEQ_2005_ varies between 3.1 and 7.2 pg TEQs/g fat for the three countries studied in Africa (UNEP 2009). The use of mean or median concentrations applying TEF_1998_ or TEF_2005_ results in four different total TEQs. Still, some spatial comparisons are possible. Both Brazil (Paumgartten et al. 2000) and Chile (UNEP 2009) show higher total TEQ_2005_ than the African countries. When comparing these total TEQ_2005_ concentrations with mothers’ milk from Europe (Table 6), we confirm generally higher TEQs from Europe than the countries mentioned in Africa and South America. Further comparisons are not made here due to the extensive complications in doing so.

### PBDEs

**Fig. 7:**
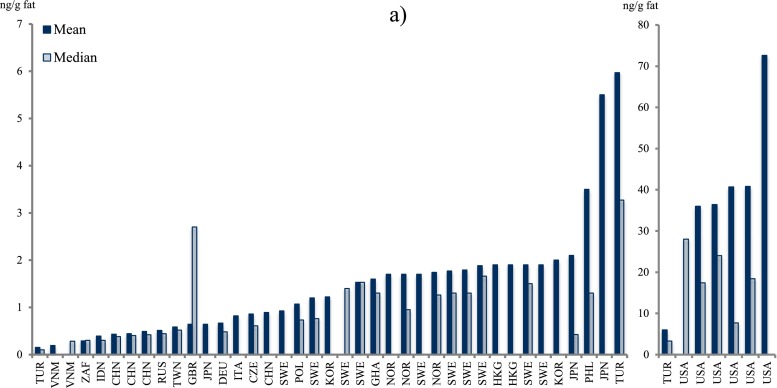
Graphical presentation of BDE-47 concentrations in mothers’ milk from countries worldwide

**Fig. 8:**
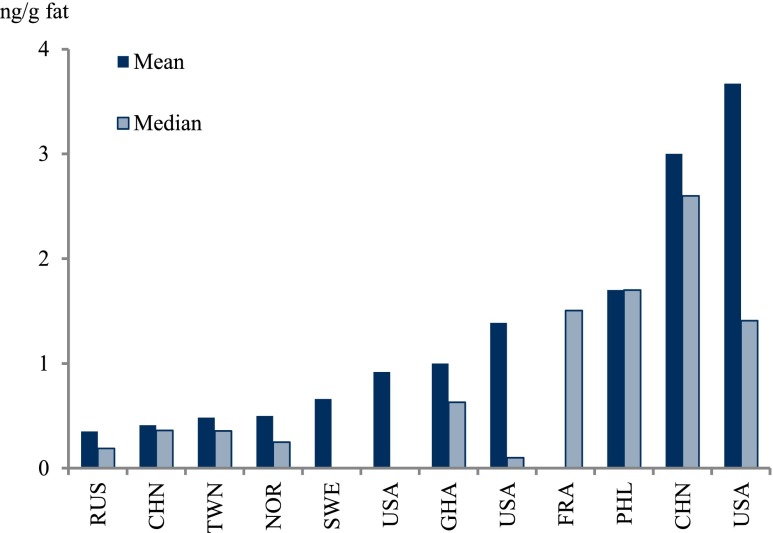
Graphical presentation of BDE-209 concentrations in mothers’ milk from countries worldwide.

Concentrations of PBDEs, BDE-47, BDE-209, and ∑PBDE data are summarized in Table 7 and Figs. 7 and 8.

#### Africa

Only two studies of PBDEs in mothers’ milk from Africa were identified, and both studies confirm the occurrence of BDE-47 in mothers’ milk at mean or median concentrations below 2 ng/g fat (Asante et al. 2011; Darnerud et al. 2011). BDE-209 was only reported in one study from Ghana (Asante et al. 2011).

#### Asia and the Pacific region

The concentrations of BDE-47 are rather uniform and low (below 2 ng/g fat) in mothers’ milk in Asia and the Pacific region, even though a very large geographical area is covered. One study from Japan reports the highest level of BDE-47 outside the USA with a mean concentration of 19 ng/g fat (Akutsu and Hori 2004). From the Philippines, a study reports somewhat elevated levels, mean concentrations of 1.2 and 4.9 ng/g fat (13). Both low and high BDE-47 concentrations have been determined in samples from Turkey, with mean concentrations reaching 6.0 ng/g fat (Erdogrul et al. 2004; Ozcan et al. 2011).

BDE-209 was reported in 6 out of 16 studies with similar levels (<1 ng/g fat) independent of the study (cf. Table 7). The highest BDE-209 concentrations are reported in mothers’ milk from the Philippines and Vietnam with levels of around 2 ng/g fat (Malarvannan et al. 2009; Tue et al. 2010).

The reported ∑PBDEs in Asian mothers’ milk confirm the observations of BDE-47 concentrations, with the highest concentrations from the Philippines, mean concentrations of 5–10 ng/g fat (Malarvannan et al. 2009; Sudaryanto et al. 2008b), as well as from Japan and Turkey with mean concentrations of 31 and 67 ng/g fat, respectively (Akutsu and Hori 2004; Ozcan et al. 2011). These concentrations are comparable with the levels reported in mothers’ milk from the USA.

#### Europe

In general, the concentrations of BDE-47 in mothers’ milk in Europe (approximately 1–2 ng/g fat) are higher compared to the levels in Asia and the Pacific region and Africa but lower than in the Americas. The lowest levels of BDE-47 in Europe are reported in samples from the Czech Republic, Germany, Italy, and Russia, with mean concentrations below 1 ng/g fat (Alivernini et al. 2011; Kazda et al. 2004; Polder et al. 2008a; Raab et al. 2008). The highest levels are reported in mothers’ milk from the UK with median concentration of 2.7 ng/g fat (Kalantzi et al. 2004), and the remaining results from Europe are in between the mentioned BDE-47 concentrations (Table 7). Hence, the differences in the levels are rather small.

As few as 7 out of 25 of the European mothers’ milk samples report BDE-209, with the highest concentrations in samples from France, 1.5 ng/g fat (Antignac et al. 2008; Athanasiadou and Bergman 2008). Since BDE-209 has a short half-life in humans, 14 days (Thuresson et al. 2006), the differences in concentrations of this PBDE congener vary greatly. Consequently, exposure levels of BDE-209 and nona-BDEs become uncertain when seen over time.

In Europe, the ∑PBDE concentrations are in general 3–4 ng/g fat, with a few exceptions (Table 7). The highest ∑PBDE concentrations are reported in mothers’ milk from the UK, with median levels of 6.3 ng/g fat, which is still three to five times lower than levels reported from the USA.

#### The Americas

Only studies from the USA could be found that report levels of PBDEs from the Americas and that met the criteria set for this review.

In general, the reported levels of BDE-47 are much higher in the mothers’ milk samples from the USA compared to the rest of the world. The concentrations are rather uniform, with mean values at 35–40 ng/g fat (Johnson-Restrepo et al. 2007; Schecter et al. 2003, 2005, 2010), but with levels reaching as high as 73 ng/g fat (Park et al. 2011). Also, the levels of BDE-209 are higher in the USA than the rest of the world (Table 7), which is indicating a higher prevalence of deca-BDE exposure.

Also, the concentrations of ∑PBDEs in the samples from the USA are overall similar, with means of 66–76 ng/g fat, and one median value of 51, reported in four different studies (Johnson-Restrepo et al. 2007; Schecter et al. 2003, 2005, 2010). However, one study reports a mean concentration as high as 130 ng/g fat (Park et al. 2011).

The results clearly show that US mothers’ milk contains the highest concentrations of PBDEs. This is in line with any other exposure study from the USA, showing mothers and other individuals being subjected to environmental exposures of PBDEs that are the highest in the world.

### Heptachlor

Heptachlor concentrations reported as *cis*-HCL-epoxide, HCL-epoxide, and heptachlor from 49 studies were selected and tabulated (Table 8). Concentrations of *cis*-HCL-epoxide are only reported from one or a few countries from each continent/region, and they range between 0.7 ng/g fat in China (Hedley et al. 2010) and 8 ng/g fat in Brazil (Paumgartten et al. 2000).

#### Africa

Heptachlor was only reported from four regions of the African continent with concentrations ranging between 0.9 and 2.25 ng/g fat (UNEP 2009, 2011).

#### Asia, Australia, and the Pacific region

Fifteen studies report concentrations of HCL-epoxide. Most of these report concentrations below 10 ng/g fat, but higher concentrations are reported from Australia, 53 and 78 ng/g fat (Quinsey et al. 1995); Jordan, 190 ng/g fat (Nasir et al. 1998); Thailand, 60 ng/g fat (Stuetz et al. 2001); and Turkey, 61 ng/g fat (Cok et al. 2005). The highest reported concentration of heptachlor is from Jordan at 500 ng/g fat (Nasir et al. 1998).

#### Europe

Concentrations of HCL-epoxide reported from Europe are in most cases below or close to 3 ng/g fat, although higher concentrations are reported from Ukraine, 16–22 ng/g fat (Gladen et al. 1999, 2003), and the Netherlands, 30 ng/g fat (Albers et al. 1996).

#### The Americas

Concentrations of HCL-epoxide and heptachlor from the Americas are reported as less than 4 ng/g fat except for Mexico, 160 and 580 ng/g fat, respectively (Rodas-Ortiz et al. 2008).

### Dieldrin, endrin, and aldrin

The OCPs discussed herein were regulated at an early stage in many countries. Despite of this, rather high concentrations of dieldrin are reported (Table 9).

#### Africa

No studies were retrieved that report aldrin and/or endrin in mothers’ milk from any African nation. Dieldrin was reported in seven studies, three of which were part of the UNEP screening program with the lowest levels in mothers’ milk from Ghana, Nigeria, and Senegal, with mean concentrations of 1.3–4.1 ng/g fat (UNEP 2009). However, some reports indicate levels of up to 25–120 ng/g fat (Ennaceur et al. 2007, 2008; Ntow et al. 2008).

#### Asia and the Pacific region

Although only few studies report aldrin concentrations in mothers’ milk, the variation is great. The most comprehensive study originates from Australia showing a large national spatial distribution in a range 0.01–0.68 ng/g fat (Harden et al. 2007). Somewhat higher levels of aldrin are found in Kuwait and the highest concentration are from India and Jordan with mean concentrations up to 860 ng/g fat (Nasir et al. 1998; Siddiqui et al. 2002).

Dieldrin is more frequently reported in mothers’ milk than aldrin and endrin and at rather high concentrations (Table 9). The levels of dieldrin are rarely above 100 ng/g fat, with the exceptions of one study from Jordan, which reports a mean concentration of 1400 ng/g fat (Nasir et al. 1998), and one study from China, 9300–10,000 ng/g fat (Wang et al. 2008). Only two studies have been retrieved reporting on endrin in mothers’ milk, one from Jordan and one from Kuwait (Nasir et al. 1998; Saeed et al. 2000).

#### Europe

A study from Croatia reports aldrin and endrin in their samples, with mean concentrations of 1.3 and 2.0 ng/g fat, respectively (Frkovic et al. 1996). Again, the occurrence of dieldrin in mothers’ milk is frequently reported from European countries in low concentrations, for example in samples from Germany and Croatia, with mean concentrations below 3.8 ng/g fat (Frkovic et al. 1996; Zietz et al. 2008). However, other studies from Germany show higher levels, with mean concentrations of 4 and 14 ng/g fat (Raab et al. 2008; Schlaud et al. 1995). Two reports investigating dieldrin levels in mothers’ milk, both in Denmark and Finland, show similar results (Shen et al. 2007, 2008) and likewise from the WHO-UNEP monitoring program (UNEP 2011). The highest levels are reported from the UK with a mean concentration of 48 ng/g fat (Harris et al. 1999) and the Netherlands with a median concentration of 50 ng/g fat (Albers et al. 1996).

#### The Americas

In the Americas, one study has reported levels of aldrin from a known pest-controlled area on the Yucatán peninsula in Mexico with a mean concentration of 280 ng/g fat (Rodas-Ortiz et al. 2008). In the lower end of reported levels of dieldrin in mothers’ milk are samples from the Americas, Antigua and Barbuda, Chile, and Uruguay, with mean concentrations of 2.6, 5.0, and 4.9 ng/g fat (UNEP 2009), respectively, as well as from the Group of Latin American and Caribbean countries, with a median concentration of 4.9 ng/g fat (UNEP 2011). The highest levels of dieldrin in mothers’ milk in the Americas are found in samples from a know pest-controlled area in on the Yucatán peninsula in Mexico with a mean concentration of 300 ng/g fat (Rodas-Ortiz et al. 2008). Endrin could only be found in a sample from Nicaragua (Romero et al. 2000) and from the abovementioned pest-controlled area on the Yucatán peninsula in Mexico (Rodas-Ortiz et al. 2008).

### Pentachlorobenzene, toxaphene, and mirex

Pentachlorobenzene (PCBz), mirex, and toxaphene concentrations in mothers’ milk are only reported in a few studies (Table 10). Mirex levels are reported as 16, 20, and 68 ng/g fat in mothers’ milk from Germany, Russia, and Canada, respectively (Newsome and Ryan 1999; Polder et al. 2008a; Skopp et al. 2002). Both PCBz and mirex are reported in around 1 ng/g fat in most mothers’ milk samples, with a few exceptions (Table 10). The most profound exception is mirex found in a concentration of 200 ng/g fat in mothers’ milk from Mexico (Rodas-Ortiz et al. 2008).

It is notable that so few reports have been published on these POPs. Therefore, any assessments of spatial differences are impossible.

### HBCDD and PBB

**Fig. 9:**
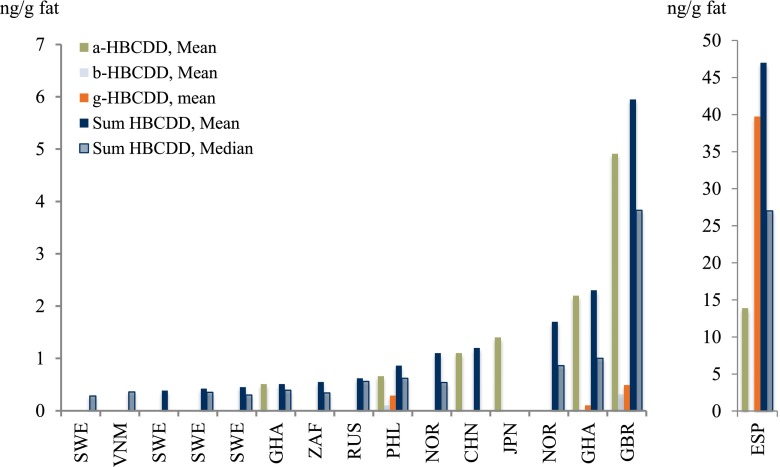
Graphical presentation of HBCDD concentrations in mothers’ milk from countries worldwide

Concentrations of hexabromocyclododecane (HBCDD) and polybrominated biphenyl (PBB) reported in mothers’ milk are presented in Table 11 on a fat weight basis. From isomer-specific information, it is clear that α-HBCDD is the most abundant isomer of environmental HBCDDs (Eljarrat et al. 2009; Lankova et al. 2013). The spatial distribution of HBCDD is illustrated in Fig. 9.

#### Africa

Only two studies of HBCDDs in mothers’ milk from all of Africa were retrieved, both showing subnanogram per gram fat concentrations in the mothers’ milk analyzed (Asante et al. 2011; Darnerud et al. 2011). No study was found reporting on PBBs in mothers’ milk from any African nation.

#### Asia and the Pacific region

The lowest concentrations of ∑HBCDDs reported in mothers’ milk from Asia come from the Philippines and Vietnam with mean and median concentrations below 1 ng/g fat (Malarvannan et al. 2009; Tue et al. 2010). A study from China shows a greater range in ∑HBCDD concentrations, with means of 0.33–2.8 ng/g fat (Shi et al. 2009). In Japanese mothers’ milk, ∑HBCDDs levels of 1.4 ng/g fat (Kakimoto et al. 2008) are reported. No study was found reporting on PBBs in mothers’ milk from any nation in Asia or the Pacific region.

#### Europe

The levels of ∑HBCDDs in Europe are quite uniform and low (Table 11), i.e., <1 ng/g fat. However, the highest concentrations on a global scale are those reported from a Spanish study, with a mean concentration of 47 ng/g fat, in mothers’ milk from a population living close to a textile processing plant (Eljarrat et al. 2009). The ∑HBCDD concentrations in Swedish samples reported median concentrations within the range of 0.3–0.4 ng/g fat (Glynn et al. 2011). Somewhat higher concentrations, and comparable with the Japanese levels, are reported from Norway, with mean concentrations of 1.7 ng/g fat (Thomsen et al. 2010). The ∑HBCDDs in mothers’ milk from the UK report a mean concentration of 6.0 ng/g fat (Abdallah and Harrad 2011), which is the highest background level worldwide, apart from the Spanish “hot spot” samples. One study reports PBB concentrations from two countries, Denmark and Finland, both indicating mean concentrations below 0.3 ng/g fat (Shen et al. 2008).

#### The Americas

We have not found any study with our search criteria which has reported the presence of HBCDDs or PBBs in mothers’ milk from any nation in the Americas.

### PFOS

**Fig. 10:**
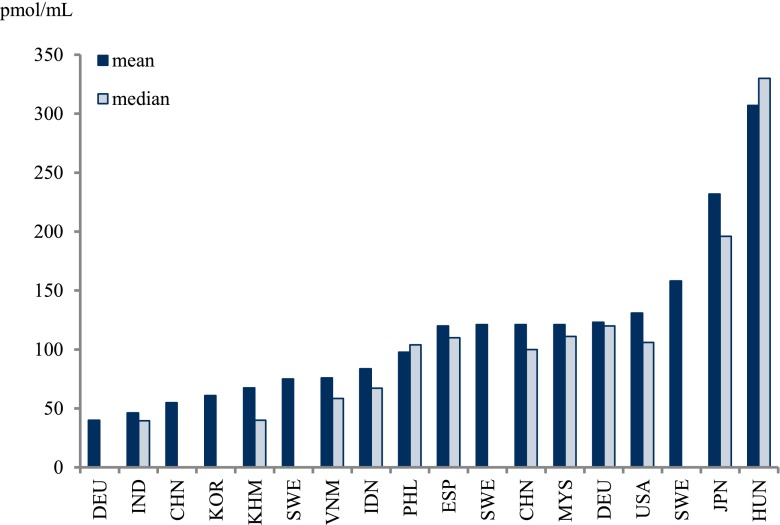
Graphical presentation of PFOS concentrations in mothers’ milk from countries worldwide

Thirteen studies report PFOS concentrations in mothers’ milk from a total of 13 countries in Asia and Europe and one study from the USA (Table 12). The concentration of PFOS ranges between 39 and 200 pg/mL, with Hungary as the only exception, 330 pg/mL (Volkel et al. 2008). Due to the limited data, it is not possible to draw any conclusions regarding spatial exposure differences. The spatial distribution of PFOS is illustrated in the diagram in Fig. 10.

### Endosulfan

Only some scattered endosulfan mothers’ milk data are available (Table 13). The sum concentrations from India and Turkey are exceptionally high, i.e., concentrations above 1000 ng/g fat (Cok et al. 2011; Sanghi et al. 2003). These high levels are comparable to some data on DDT in mothers’ milk (cf. Table 2) and are likely due to the very recent use of endosulfan in these countries.

### Chlordecone

No studies were found with our search criteria reporting chlordecone concentrations in mothers’ milk.

### SCCPs

No studies were found with our search criteria reporting SCCP concentrations in mothers’ milk, even though there is an agency report on SCCPs in Swedish mothers’ milk (Darnerud et al. 2012) indicating their presence in this matrix.

### Global distribution trends

The data collected and compiled within the study indicates that there is indeed a difference in the distribution and exposure to POPs which is dependent on where in the world one resides. These conclusions are more easily made when comparing the different spatial distribution diagrams, e.g., Fig. 3. In general, it was found that DDT/DDE pesticides were reported in higher concentrations in mothers’ milk from the regions of Africa, Asia, and Central America, with a propensity for agricultural economies and lower degree of industrialization. On the other hand, PCBs and dioxins were found to be reported to a higher degree in more industrialized regions, such as parts of Asia, Europe, and North America. A good example of this can be seen by comparing Figs. 3 and 4a–c, where the DDT/DDE concentrations clearly are lower, in general, in Europe and North America compared to the rest of the world (Fig. 3). Similarly, it can be seen from Fig. 4a–c that the PCB concentrations are higher in industrialized regions compared to the rest of the world. This pattern is also observed for HCHs, although there are a few observations of high concentrations in mothers’ milk samples from Eastern Europe, i.e., Russia, Romania, and Ukraine (Fig. 6a–d).This pattern is not surprising since PCBs and dioxins, not shown in spatial distribution diagrams, are related to a degree of industrialization, either as chemical products or impurities there within. DDT as well as HCHs has been used as pesticides in SC and has been used in the equatorial and subequatorial regions, which in general are less industrialized and more dependent on agriculture. Furthermore, it is clear that mothers’ milk from the USA contains more PBDEs than the rest of the world (Fig. 7). BDE-47 is a biomarker of PBDE exposure and the lowest reported concentration is around five times as big as the highest concentration in a sample from outside of the USA. This can be explained by the stricter flame retardant policy enforced within the USA, a policy which calls for a greater use of flame retardant substances such as PBDEs primarily in upholstery (GSPI 2013; State of California 2000, 2013). A new fire safety regulation has recently been adopted, January 1st 2014, which does not call for the use of flame retardant chemicals and perhaps this will lead to a decrease of PBDEs in mothers’ milk in the USA (GSPI 2013; State of California 2000, 2013). For the substances not mentioned, there are no observed, clear spatial distribution trends that can be explained by traditional/historical use of the substances in question. This could be since there are too few reported concentrations available or that the differences in use or emissions are too small to observe.

## Temporal trends

Two distinct objectives can be identified concerning temporal trend monitoring of contaminants. One is to quantitatively estimate the rate of changes in contaminant concentration, e.g., as a change in percent per year or as half- or doubling time in number of years. An example of this could be to estimate the response of measures taken to reduce the discharges of various contaminants. Dissimilarities in comparisons between the rate of change in contaminant concentration in mothers’ milk and other environmental biological matrices (e.g., fish) can give information about the exposure patterns, i.e., if the mothers are exposed to contaminants not only from local food but also from imported food and the indoor environment, including a variety of man-made technical products. Another objective of temporal trend monitoring is to study emerging new substances and to detect renewed use of banned contaminants. In order to estimate the rate quantitatively with a high statistical power, it is essential to keep the random variation between years as low as possible. Compared to other matrices, mothers’ milk seems to show a relatively low random variation (UNEP 2004).

Inclusion of a time series in this review article requires a minimum of five reported data points. Only approximately half of the substance groups from only two countries, Sweden and Japan, fulfilled the described criteria. The temporal trends present data from 1972 to 2011.

In the graphs below, log-linear regression and a smoother test have been carried out. The smoother test checks if the smoother explains significantly more of the variation in concentration, than the regression line (Nicholson et al. 1998).The regression line and/or the smoother are plotted when significant (*α* = 0.05).

### DDT and DDT-related compounds

**Fig. 11:**
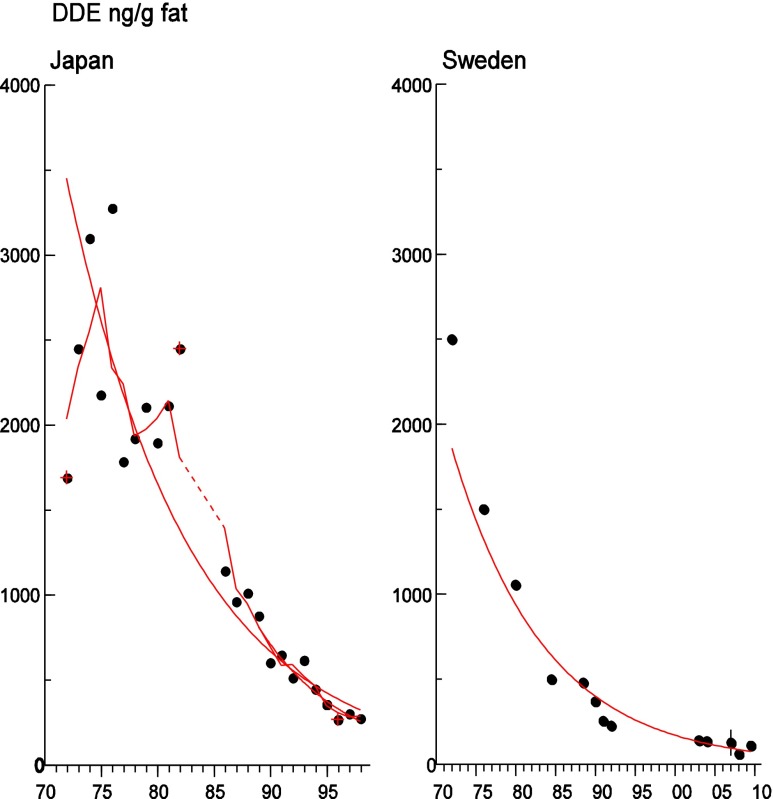
Temporal trends of DDE (ng/g fat) from Japan (Konishi et al. 2001) and Sweden (Lundén and Norén 1998; Athanasiadou and Bergman 2008; Bergman et al. 2010)

In Fig. 11, the concentrations of DDE (ng/g fat) in the samples from Japan, 1972–1998 (Konishi et al. 2001), and Sweden, 1972–2010 (Athanasiadou and Bergman 2008; Bergman et al. 2010; Lundén and Norén 1998), show significant decreasing trends over the whole time period of −9.1 % (*p* < 0.001) and −8.5 % (*p* < 0.001), respectively. The Japanese samples also show significant decreasing concentrations for the last 10 years of −13 % (*p* < 0.001), while no trend is indicated in the Swedish samples for the last 10 years (estimated during a decade later than for the Japanese samples). The temporal trends for DDT in mothers’ milk from Japan and Sweden are of similar magnitude as for DDE. In addition, the trends observed in Swedish mothers’ milk for DDE coincide with the trends seen in Swedish freshwater (Nyberg et al. 2011) and marine (Bignert et al. 2012) biota.

**Fig. 12:**
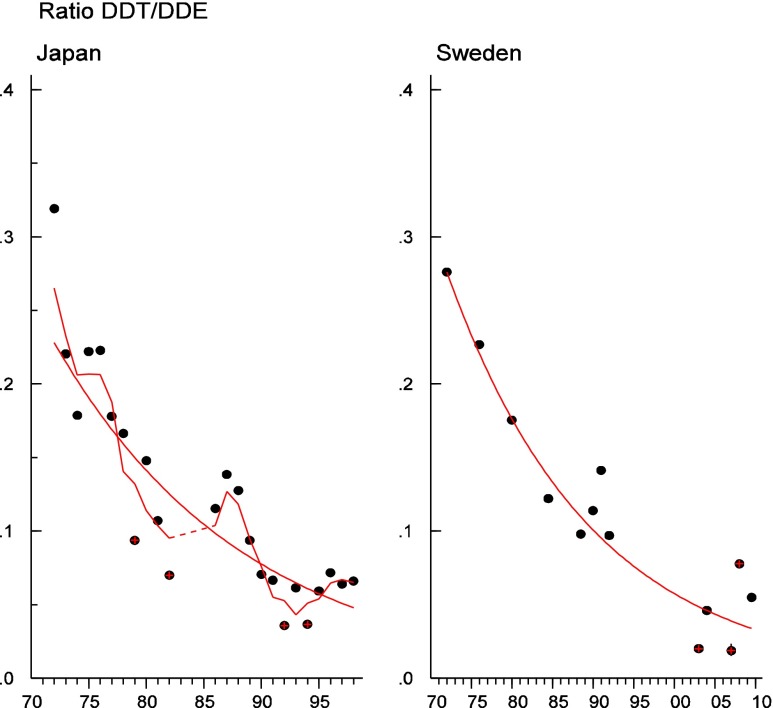
Temporal trends of the ratio between DDT and DDE (ng/g fat) from Japan (Konishi et al. 2001) and Sweden (Lundén and Norén 1998; Athanasiadou and Bergman 2008; Bergman et al. 2010)

The ratio of DDT/DDE (Fig. 12) shows similar log-linear trends in the samples from Japan, 1972–1998 (Konishi et al. 2001), and Sweden, 1972–2010 (Athanasiadou and Bergman 2008; Bergman et al. 2010; Lundén and Norén 1998). The Japanese samples also show a significant nonlinear trend for the ratio of DDT/DDE, which might indicate that a new release of DDT has occurred during the monitoring period.

### PCBs

**Fig. 13:**
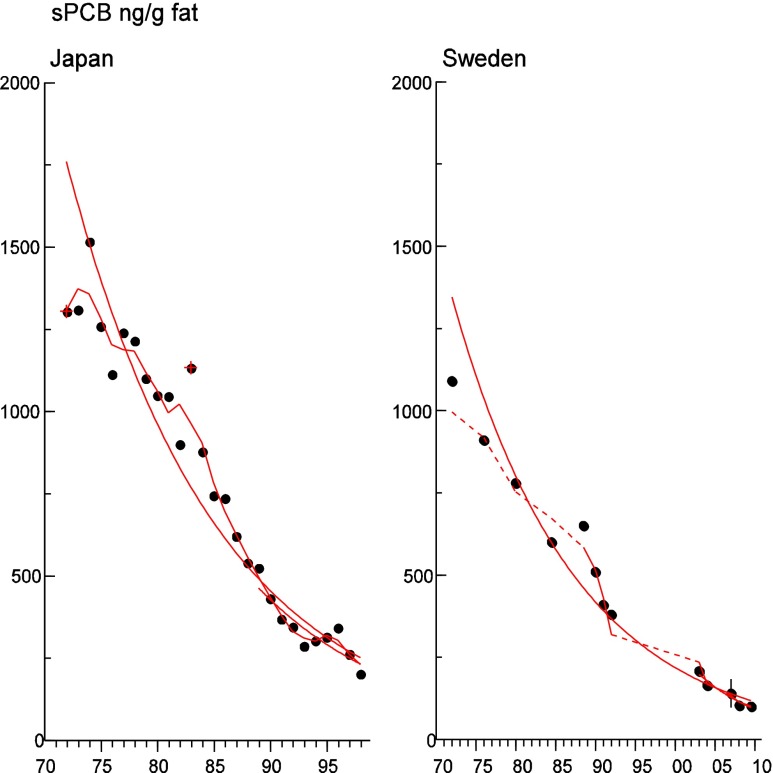
Temporal trends of ΣPCB (ng/g fat) from Japan (Konishi et al. 2001) and Sweden (Lundén and Norén 1998; Athanasiadou and Bergman 2008; Bergman et al. 2010)

In Fig. 13, the concentrations of ∑PCB (ng/g fat) in the samples from Japan, 1972–1998 (Konishi et al. 2001), and Sweden, 1972–2010 (Athanasiadou and Bergman 2008; Bergman et al. 2010; Lundén and Norén 1998), show significant decreasing trends over the whole time period of −7.5 % (*p* < 0.001) and −6.5 % (*p* < 0.001), respectively, and for the last 10 years of −7 % (*p* < 0.001) and −11 % (*p* < 0.011), respectively. The trends observed in Swedish mothers’ milk for ∑PCB coincide with the trends seen in Swedish freshwater (Nyberg et al. 2011) and marine (Bignert et al. 2012) biota.

**Fig. 14:**
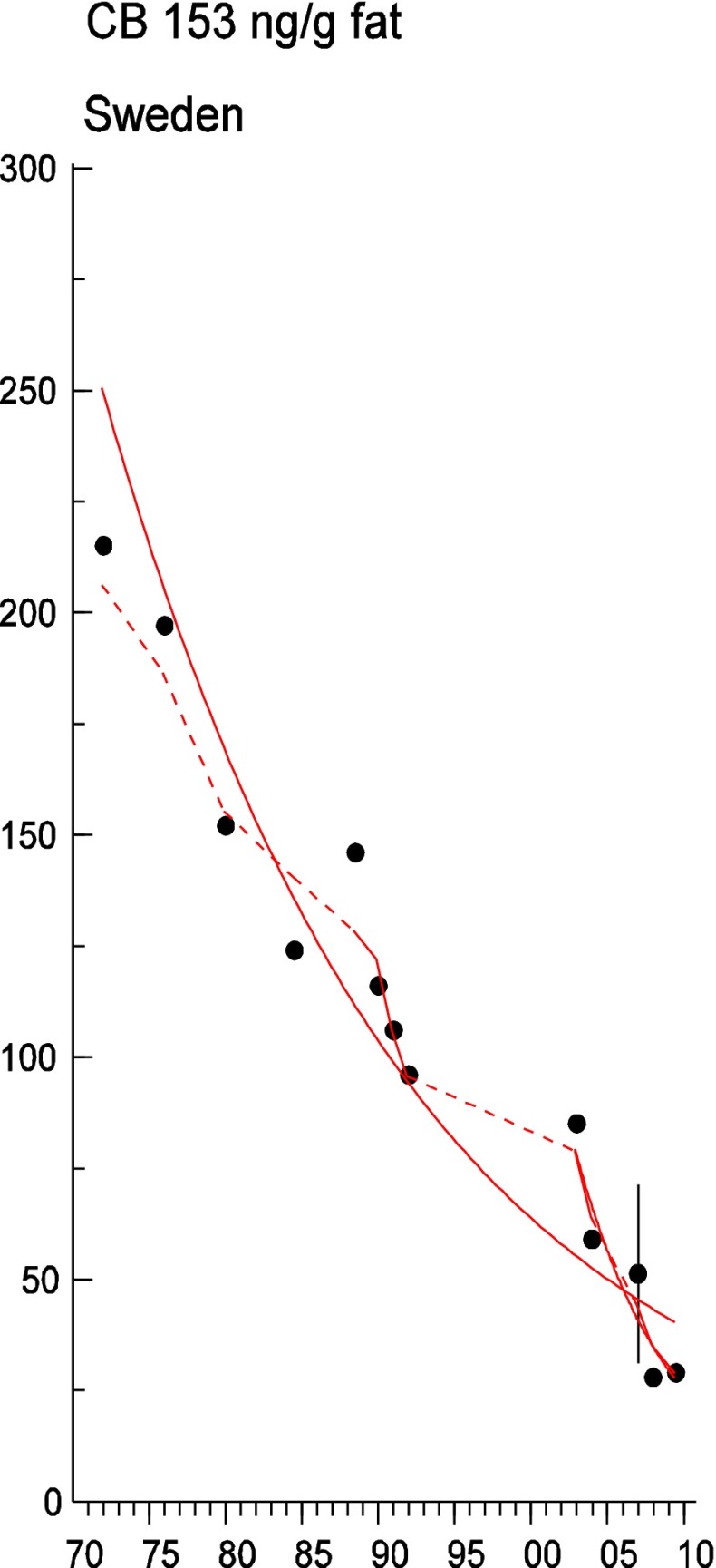
Temporal trend of CB-153 (ng/g fat) from Sweden (Lundén and Norén 1998; Athanasiadou and Bergman 2008; Bergman et al. 2010)

Only one temporal trend study on congener basis was found for PCBs within this review (from Sweden). In Fig. 14, the temporal trend for CB-153 (ng/g fat) in the Swedish samples, 1972–2010 (Bergman et al. 2010; Lundén and Norén 1998), is presented. CB-153 shows a significant decreasing trend over the whole time period and for the last 10 years of −4.9 % (*p* < 0.001) and −5.9 % (*p* < 0.042), respectively.

### HCB and HCHs

**Fig. 15:**
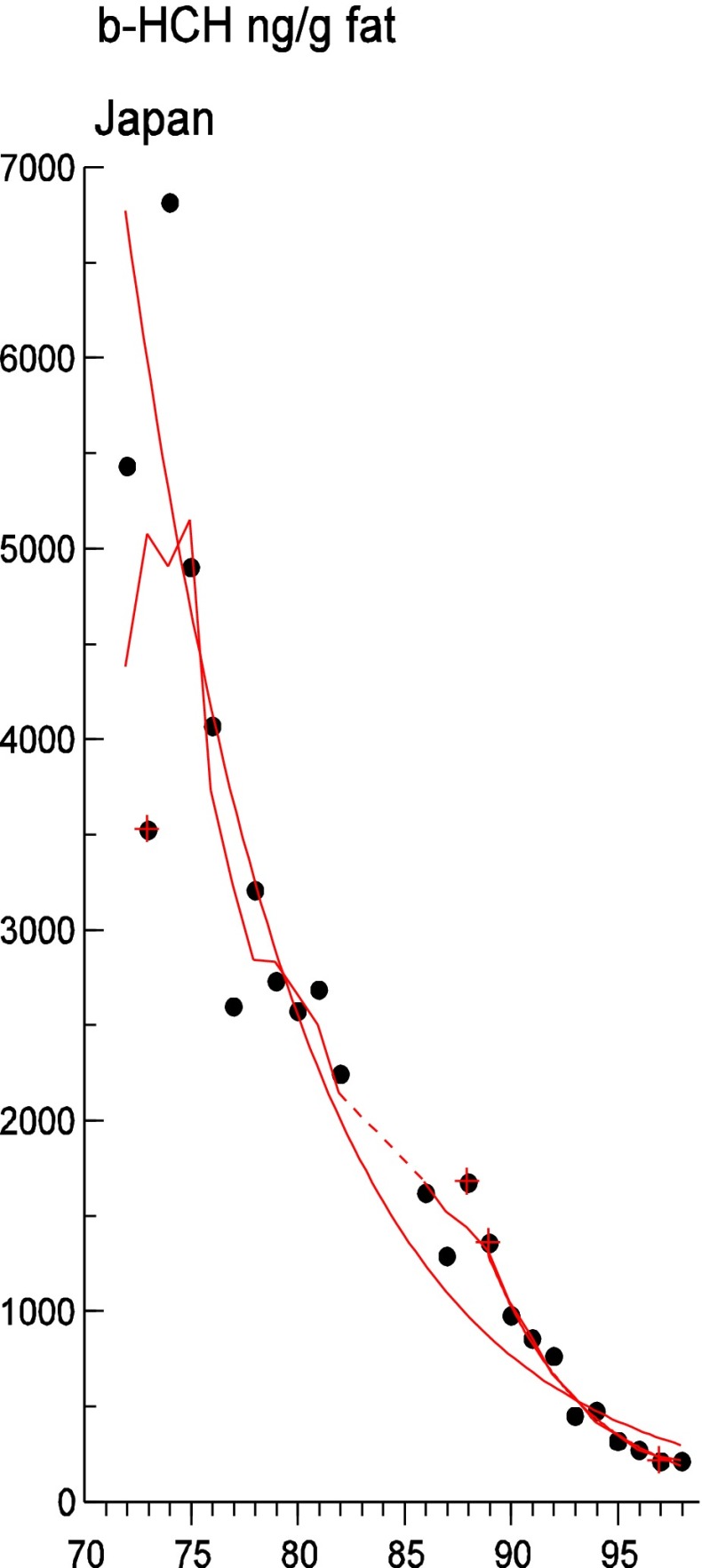
Temporal trends of β-HCH (ng/g fat) from Japan (Konishi et al. 2001)

In Fig. 15, the concentrations of β-HCH (ng/g fat) in the samples from Japan, 1972–1998 (Konishi et al. 2001), show a significant decreasing trend over the whole time period as well as for the last 10 years of −12 % (*p* < 0.001) and −11 % (*p* < 0.001), respectively.

### PCDDs, PCDFs, and DL-PCBs

**Fig. 16:**
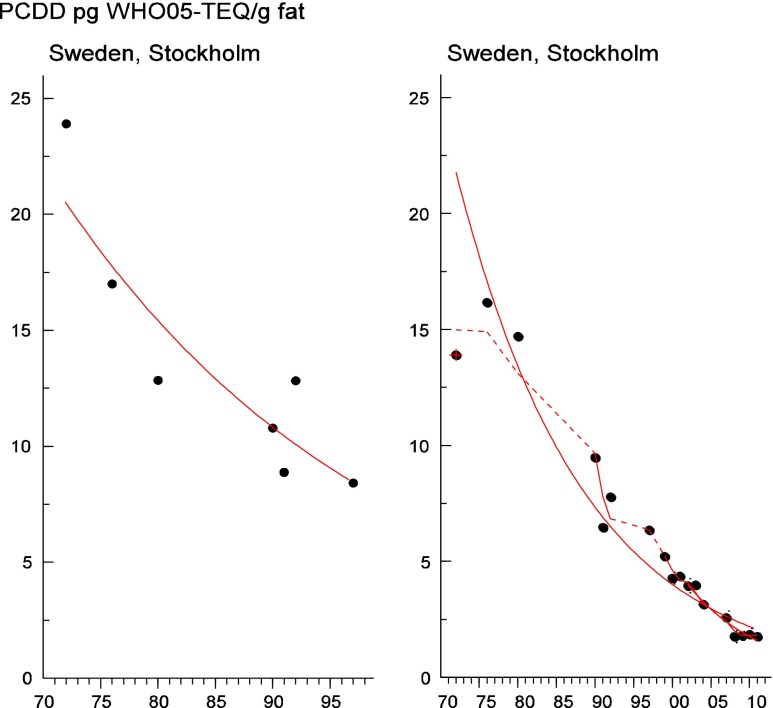
Two temporal trends of PCDD in WHO_2005_-TEQ (pg/g fat) from Sweden, on the left (Norén and Meironyte 2000) and on the right (Fång et al. 2013)

In Fig. 16, the concentrations of PCDD (pg WHO_2005_-TEQ/g fat) from Stockholm, 1972–1997 (Norén and Meironyte 2000) and 1972–2011 (Fång et al. 2013), show significant decreasing trends over the whole time period of −3.6 % (*p* < 0.005) and −6.0 % (*p* < 0.001), respectively. However, for the last 10 years, a significant decreasing trend of −10 % (*p* < 0.001) is only seen for the time series from 1972 to 2011 (Fång et al. 2013), covering the last decade in contrast to the study from 1972 to 1997 (Norén and Meironyte 2000), which might have too few samples during the last 10 years to detect a trend. It should be noted that the two studies by Norén and Meironyte and the study by Fång et al. are analyzing the same pooled mothers’ milk sample during 1972–1997.

**Fig. 17:**
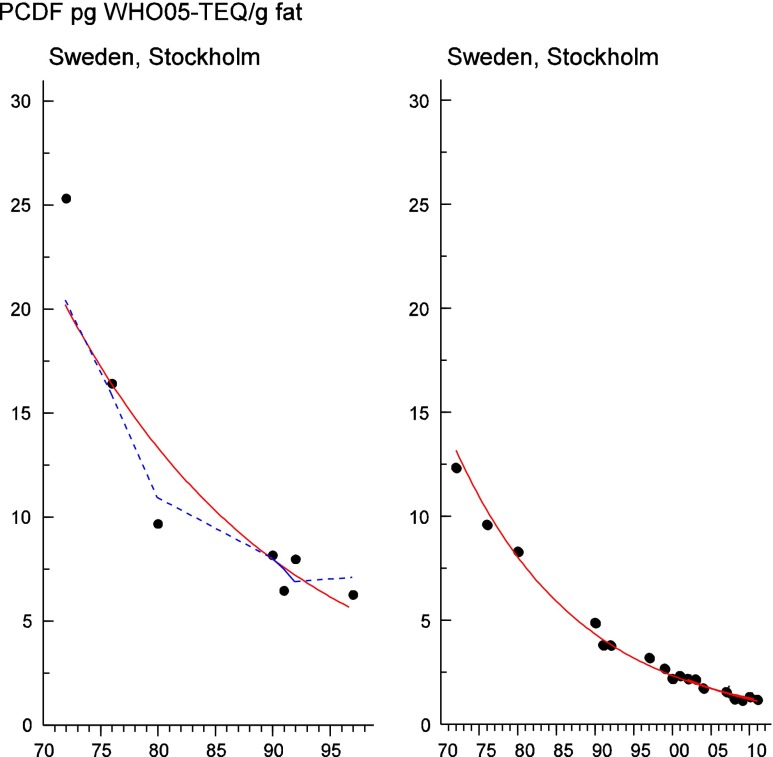
Two temporal trends of PCDF in WHO2005-TEQ (pg/g fat) from Sweden, *on the left* (Norén and Meironyte 2000) and *on the right* (Fång et al. 2013)

In Fig. 17, the concentrations of PCDF (pg WHO_2005_-TEQ/g fat) from Stockholm, 1972–1997 (Konishi et al. 2001; Norén and Meironyte 2000) and 1972–2011 (Fång et al. 2013), show significant decreasing trends over the whole time period of −5.2 % (*p* < 0.003) and −6.2 % (*p* < 0.001), respectively. However, for the last 10 years, a significant decreasing trend of −7.3 % (*p* < 0.001) is only seen for the time series from 1972 to 2011 (Fång et al. 2013), covering the last decade in contrast to the study from 1972 to 1997 (Norén and Meironyte 2000), which might have too few samples during the last 10 years (only 4) to detect a trend.

**Fig. 18:**
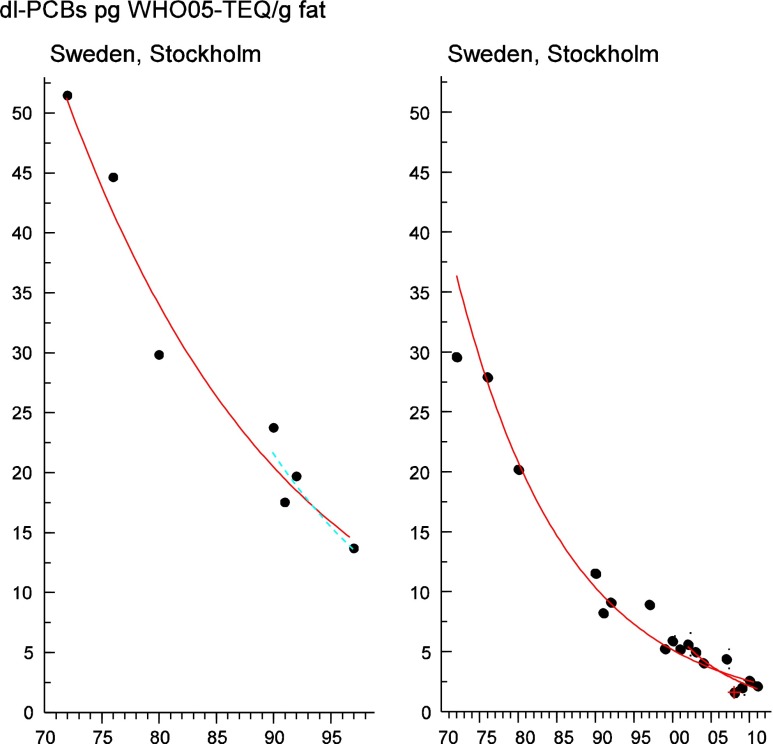
Two temporal trends of dioxin-like PCBs (DL-PCBs) in WHO_2005_-TEQ (pg/g fat) from Sweden, *on the left* (Norén and Meironyte 2000) and *on the right* (Fång et al. 2013)

In Fig. 18, the concentrations of DL-PCBs (pg WHO_2005_-TEQ/g fat) from Stockholm, 1972–1997 (Norén and Meironyte 2000) and 1972–2011 (Fång et al. 2013), show significant decreasing trends over the whole time period of −5.1 % (*p* < 0.001) and −7.0 % (*p* < 0.001), respectively. For the last 10 years, a significant decreasing trend of −12 % (*p* < 0.012) is seen for the samples from 1972 to 2011 (Fång et al. 2013), and a decreasing trend of −6.7 % (*p* < 0.107) is also indicated in the samples from 1972 to 1997 (Norén and Meironyte 2000).

The trends observed in Swedish mothers’ milk for DL-PCBs during the whole time period coincide with the trends seen in Swedish freshwater (Nyberg et al. 2011) and marine (Bignert et al. 2012) biota for the dioxin-like PCB congener CB-118 from the end of the 1970s to the beginning of the 1990s. However, during the last decade, the levels are decreasing at a higher rate in Swedish human milk than in marine and freshwater biota from Sweden.

### PBDEs

**Fig. 19:**
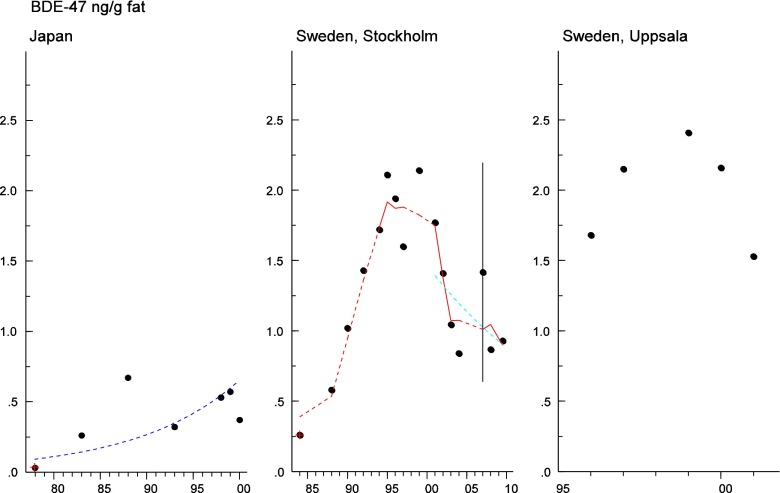
Temporal trends of BDE-47 (ng/g fat) from Japan (Akutsu et al. 2003) and from Stockholm (Athanasiadou and Bergman 2008; Fängström et al. 2008; Bergman et al. 2010) and Uppsala (Lind et al. 2003), Sweden

In Fig. 19, the concentrations of BDE-47 (ng/g fat) in the samples from Japan, 1977–1999 (Akutsu et al. 2003), indicate an increasing trend over the whole time period of 9.1 % (*p* < 0.081). In contrast, a decreasing trend of −5.7 % (*p* < 0.093) is indicated in the Swedish samples from Stockholm (Athanasiadou and Bergman 2008; Bergman et al. 2010; Fängström et al. 2008) for the last 10 years of the study. This decreasing trend of BDE-47 coincides with the trends seen in Swedish marine (Bignert et al. 2012) and freshwater (Nyberg et al. 2011) biota over the last decade. PentaBDE was first phased out voluntarily by the industry in Germany in 1986 and end in Sweden in 1999 (Alcock and Busby 2006). Subsequently, BDE-47 was partially banned within the EU countries in 2004 and the declining concentrations in human milk and biota could to some extent be explained by these events. No trend could be observed in the Swedish time trend from Uppsala during 1996–2001 (Lind et al. 2003).

### Heptachlor

**Fig. 20:**
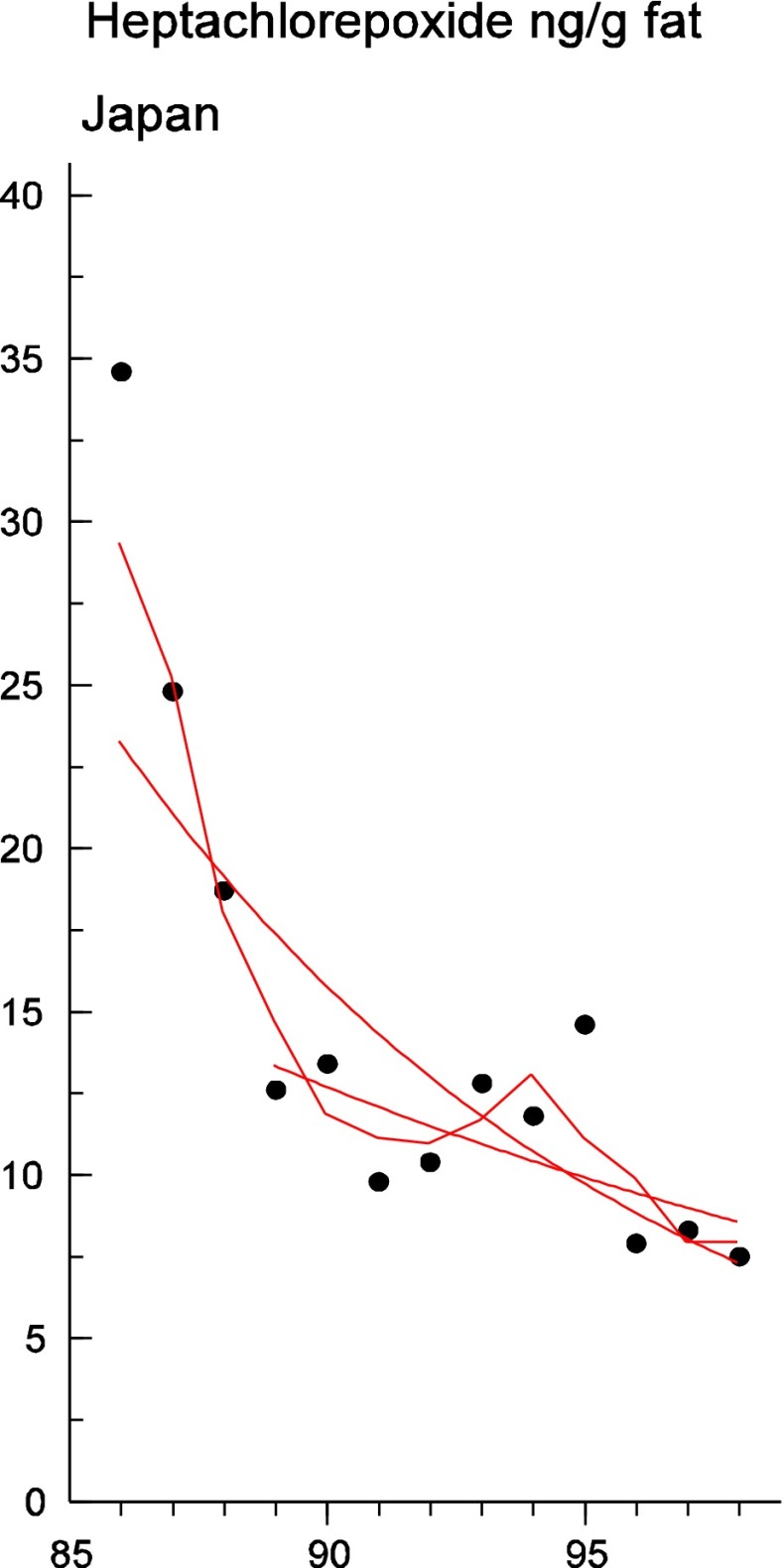
Temporal trend of heptachlorepoxide (ng/g fat) from Japan (Konishi et al. 2001)

In Fig. 20, the concentrations of heptachlorepoxide (ng/g fat) in the samples from Japan, 1986–1998 (Konishi et al. 2001), show significant decreasing trends over the whole time period and for the last 10 years of −9.7 % (*p* < 0.001) and −4.9 % (*p* < 0.049), respectively.

### Dieldrin, endrin, and aldrin

**Fig. 21:**
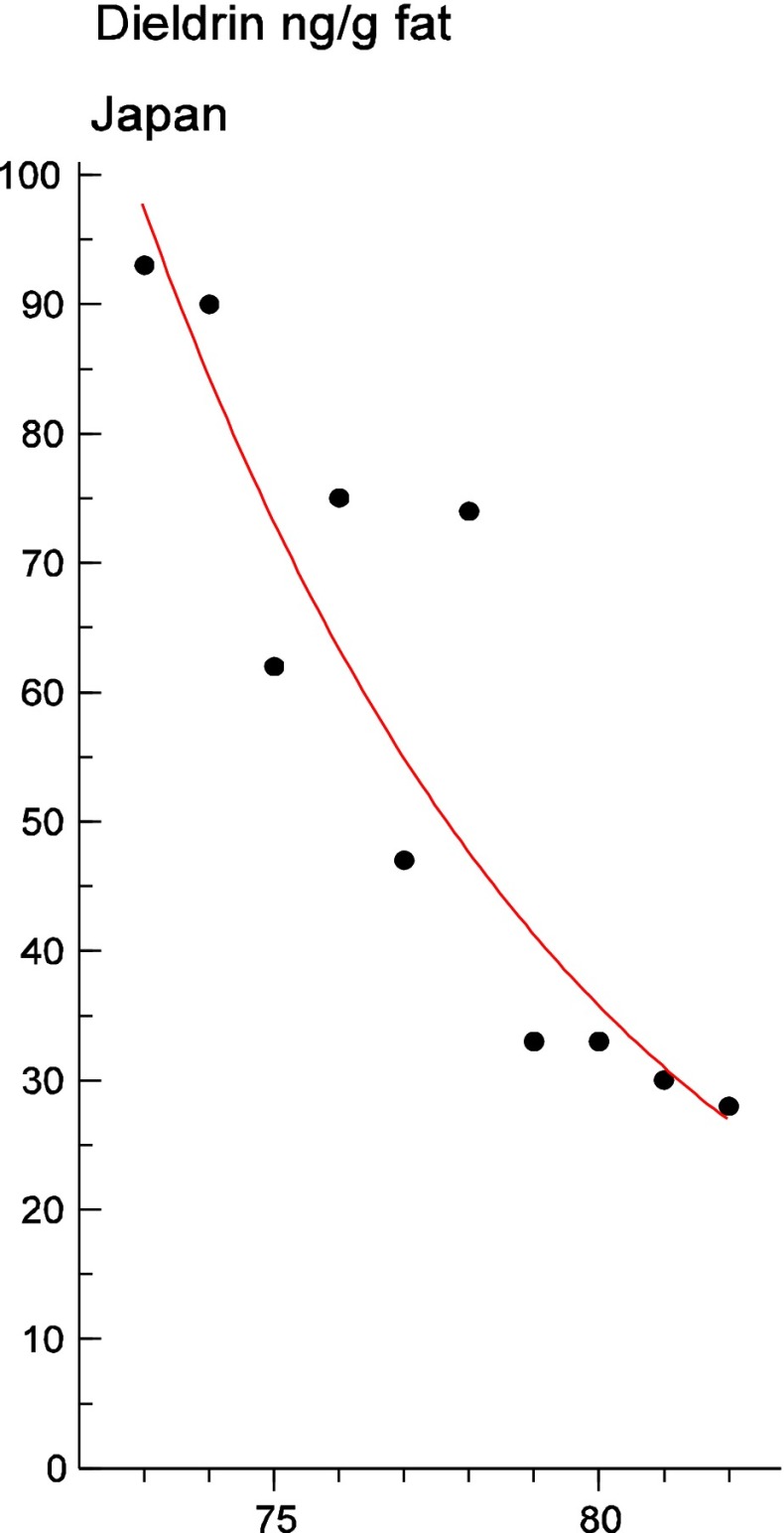
Temporal trend of dieldrin (ng/g fat) from Japan (Konishi et al. 2001)

In Fig. 21, the concentrations of dieldrin (ng/g fat) in the samples from Japan, 1972–1982 (Konishi et al. 2001), show a significant decreasing trend over the whole time period of −14 % (*p* < 0.001).

### HBCDD

**Fig. 22:**
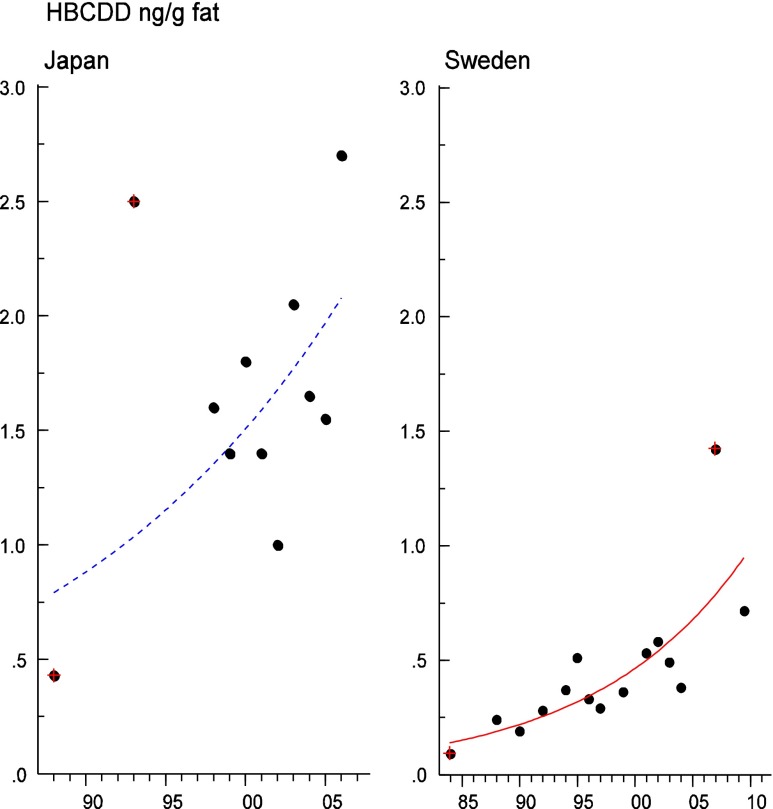
Temporal trends of HBCDD (ng/g fat) from Japan (Kakimoto et al. 2008) and Sweden (Athanasiadou and Bergman 2008; Fängström et al. 2008; Bergman et al. 2010)

The concentrations in Fig. 22 of HBCDD (ng/g fat) in the samples from Japan, 1987–2007 (Kakimoto et al. 2008) and Sweden, 1987–2010 (Athanasiadou and Bergman 2008; Bergman et al. 2010; Fängström et al. 2008), show increasing trends over the whole time period of 5.4 % per year (*p* < 0.061) and 7.6 % per year (*p* < 0.001), respectively. The increasing trend of HBCDD seen in the Swedish milk samples coincides with trends in Swedish marine biota from the Baltic Sea (Bignert et al. 2012). HBCDD is still in use within the EU but is listed in REACHs authorization list as substance of very high concern (SVHC) and, since November 2014, included in the SC.

### PFOS

**Fig. 23:**
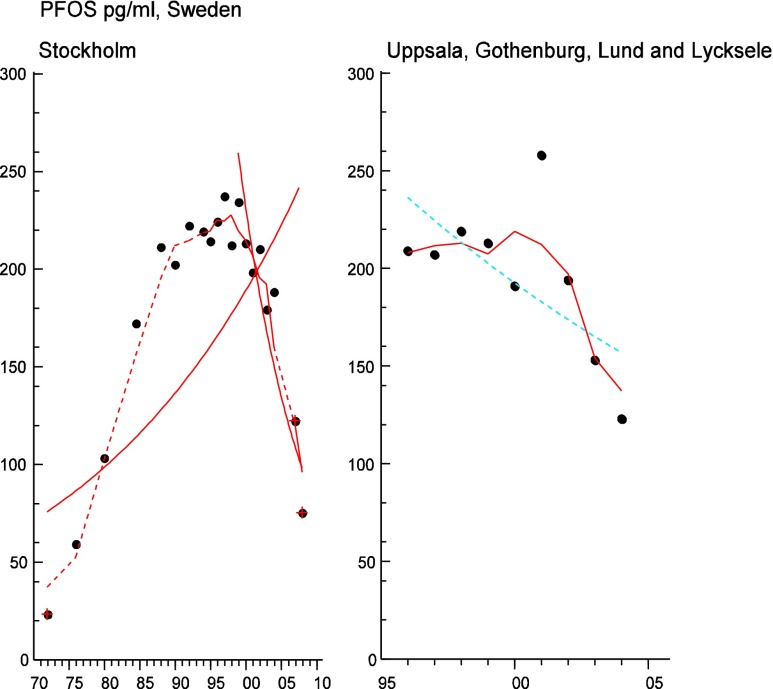
Two temporal trends of PFOS (pg/mL) from Stockholm (Sundström et al. 2011) and Uppsala, Gothenburg, Lund and Lycksele (Kärrman et al. 2007), Sweden

In Fig. 23, the concentrations of PFOS (pg/ml) in the samples from Stockholm, 1972–2008 (Sundström et al. 2011), show a significant increasing trend over the whole time period of 3.3 % (*p* < 0.012). In contrast, a significant decreasing trend of −11 % (*p* < 0.002) is observed for the last 10 years. In the study with samples from Uppsala, Gothenburg, Lund, and Lycksele, 1996–2004 (Kärrman et al. 2007), a decreasing trend is indicated (*p* < 0.059) for the whole time period. The trends observed in Swedish mothers’ milk for PFOS coincide with the trends seen in Swedish marine (Bignert et al. 2012) and freshwater (Nyberg et al. 2011) biota over the last decade.

## Critical remarks and conclusions

Some of the legacy POPs are the most well-researched environment pollutants among all. This is due to their global distribution and occurrence in humans and wildlife, being classified as CMRs, having endocrine disruption effects and/or having other toxic effects. Some early efforts focused on the transfer of these chemically stable and bioaccumulative compounds to nursing children. The transfer of POPs via mothers’ milk initiated a still ongoing debate on risks for the newborn babies. In contrast to the strict recommendations to nursing mothers to avoid smoking and drinking alcohol, it is not possible to change the mothers’ body burdens of POPs as their levels of these compounds have been built up over the mothers’ whole lifetime. However, in some countries, there are at least dietary recommendations on how to limit the intake of POPs from some major food sources, in particular fatty fish. These recommendations are primarily targeting young women and women in child-bearing ages. Due to the many positive effects of nursing, WHO recommends mothers to breast feed their newborns for a minimum of 6 months (WHO and UNICEF 2014).

WHO also initiated a monitoring program for POPs in mothers’ milk in 1976 (WHO 2009), but at this point, researchers had already started to do exposure studies of nursing infants, as reviewed by Norén and Meironyte (2000). In the present review, it becomes clear how abundant mothers’ milk is a matrix for POP analysis, although only a limited number of POPs are assessed, i.e., 7 of the 24 (HCHs counted individually) POPs in Table 1 contribute with 80 % of the exposure studies discussed herein. There are no studies covering all, or even a majority of the POPs in the same study. The most comprehensive results for POP exposure analyses are when the same milk samples are utilized for assessments of as many POPs as possible. In reality, it is much more common with scattered studies globally, regionally and country-wise. This means that the cohorts are defined by different means and the objectives vary. This leads to the conclusion that there are very few studies that allow reliable comparisons, e.g., the temporal trend studies from Japan and Sweden. However, the temporal trend studies from Japan and Sweden presented did not cover POP analyses in an optimal manner, i.e., several of the POPs were not included in these studies. Another issue regarding comprehensive time trend analysis is the need to establish environmental specimen banks whose goal is to collect and store environmental relevant samples, including mothers’ milk, in a structured manner. In time, samples collected will allow a high qualitative time trend analysis on current POPs and currently unknown pollutants. Further, this is only the start of the problem comparing POPs in mothers’ milk from around the world. Below, we list and shortly discuss some of the major shortcomings in some studies which, if rectified, would allow proper comparisons of POPs in mothers’ milk.

### Reporting base for POP concentrations in mothers’ milk

This is relevant for all POPs that are produced, used, and/or occur as mixtures of halogenated homologues and isomers-congeners. These POPs are PCBs, PCDFs, PCDDs, PBDEs, PBBs, chlordanes, toxaphen, and CPs. HCHs may be included even though only isomers of hexachlorinated cyclohexane is included. Similarly, HBCDD has three diastereomers (α-, β-, and γ-HBCDD), which are commonly discussed and often reported individually. DDTs and endosulfan, on the other hand, are both produced as two main isomers. However, both 4,4′-DDT and 2,4′-DDT are transformed to and occur in the environment, including mothers’ milk, as the corresponding DDD and DDE compounds. This has led to a similar handling of the DDTs as of true congeners of, e.g., PCBs. For all of these compounds/compound classes, it is common to report concentration sums (e.g., sPCB or ∑PCB), sometimes indicating how many PCB congeners are included in the sum and presented as ∑PCB(7), indicating that seven congeners were included. However, highly variable sums are reported for the POPs. For PCBs, for example, a number of different sum values have been found in the review including 3, 4, 6, 7, 8, 12, 15, 16, 19, 32, and 35 PCB congeners. Sometimes, the reported sum concentrations are referred to as “total PCBs” without any further specification. However, it is clear that it is still a summation of a defined number of PCB congeners, i.e., those quantified. To further complicate the issue, PCBs can also be reported as DL-PCBs and non-DL-PCBs or as non-*ortho*-PCBs, mono-*ortho*-PCBs, and di-*ortho*-PCBs. Hence, it is realistically not possible to compare ∑PCB concentrations unless they are reported in a similar manner. In this review article, we have used ∑PCB data but these are the weakest, the most unreliable, while the ∑PCB(6) and CB-153 concentration data are comparable if reported similarly by other means (mean, median, fat or fresh weight basis, cf. below).

The concentration reports for PBDEs follow a similar pattern as for the PCBs, often reporting ∑PBDEs but with differences in number and identity of PBDE congeners. Still, we have chosen to show ∑PBDE levels in Table 7, but for the purpose of comparison, BDE-47 and BDE-209 are recommended for use. The reporting variability applies for all POPs that have isomers and congeners and for which individual reference standards for analysis are available. The latter allows congener-, or isomer-, specific analysis, but the complexity of data generated call for simplifications.

Two classes of POPs, DDTs and dioxins, require further attention regarding concentration reports. The DDTs are commonly reported as the sum of 4,4′-DDT and its metabolites, 4,4′-DDE (major transformation product) and 4,4′-DDD. However, the ∑DDT may also include the true isomers, 2,4′-DDT and 4,4′-DDT or even the two isomers plus their metabolites. Concentrations of the abundant compounds 4,4′-DDT, 4,4′-DDE, and 4,4′-DDD are however quite frequently reported. This allows proper comparisons of the concentrations and to calculate comparable ratios of DDT versus either DDE or DDE and DDD, which can be done using different methods. In Table 2, we have applied the ratio 4,4′-DDT/4,4′-DDE due to the abundance of individual concentration data for these two pollutants.

The reporting of dioxin concentrations is another problem, even though individual concentration data are generated from the chemical analyses. The actual concentrations of dioxins (PCDDs, PCDFs, and DL-PCBs) are commonly reported as sum of their TEQs, after recalculation of the concentrations utilizing the TEF values—the most commonly used nowadays are the WHO TEFs from 1998 and 2005 (Van den Berg et al. 1998, 2006). However, if the ∑PCDDs, ∑PCDFs, and ∑DL-PCBs, or worse ∑dioxins, are presented, it is strongly limiting any comparisons, unless the actual concentration data of the individual congeners are presented as well.

In conclusion, POPs in mothers’ milk, as well as in other matrices, must be reported on a congener- or isomer-specific basis to promote proper trend studies. Unfortunately, this is not done in a structured manner today, which is strongly hampering the comparisons in the present data set on POPs in mothers’ milk.

### Concentration base for POPs in mothers’ milk

**Fig. 24:**
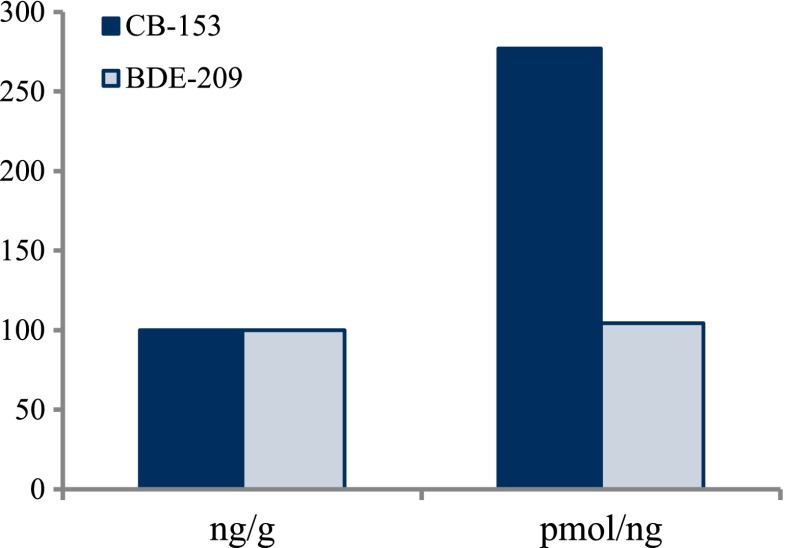
Comparison between CB-153 and BDE-209 on a weight (ng/g) and molar (pmol/g) basis

The most common way of reporting concentrations of POPs in human matrices and wildlife is on weight basis, i.e., microgram, nanogram, picogram per weight of the matrix (gram or kilogram), or on volume (e.g., mL) of the matrix, which relates to a fresh weight, volume, or extracted lipids/fats. The concentrations are rarely reported on a molar base (e.g., nmol/g or pmol/g). Despite the fact that this is the correct way of assessing exposures used for risk assessments and for correct comparisons, this means reporting is only found in very few studies of POPs in mothers’ milk. This problem is particularly evident for the polybrominated pollutants, where the molecular weight varies greatly between the different congeners. The implications are shown in Fig. 24, where it is clear that the “number of molecules” (molar base) of CB-153 is 2.5 times the number of BDE-209, although the masses of the two are equal. Since there is such an extensive span in molecular masses among the POPs, it is crucial that this must be considered for future studies/reports on POPs.

Another obstacle for comparisons of POPs in mothers’ milk is how to handle more water-soluble POPs, e.g., PFOS, other perfluorinated compounds, and organohalogen phenols. PFOS is commonly reported on a fresh weight basis, i.e., picograms per milliliter, while other POPs are reported on a fat weight basis (e.g., ng/g fat). To allow conversions, it is necessary to know the fat content of the matrix analyzed.

In conclusion, a change from weight- to molar-based reporting on POPs is needed in order to avoid unnecessary errors in exposure assessments and to allow accurate trend analyses. Furthermore, the fresh weight of mothers’ milk samples as well as concentrations on fat weight basis, and vice versa, should be mandatory since this would facilitate comparisons between studies.

### Reporting

The reported measure of central tendency of the concentrations of POPs is not consistent, i.e., the arithmetic mean, the geometric mean, or median values, and sometimes, only a range is given without a mean or a median. This hampers the possibilities to compare data. However, if a log-normal distribution can be assumed, which is common for contaminant data (see, e.g., Esmen and Hammad 1977), the geometric mean and the median can be considered equal. The arithmetic mean is, with the same assumption of log-normality, always higher than the median. Some guidance of how to adjust for this bias is given by Caudill (2010, 2012). In conclusion, commonly agreed upon guidelines for this part in reporting exposure data are also required.

### Overall conclusions

Unfortunately, reporting of POPs in mothers’ milk differs greatly between the studies. This has limited the comparisons for both spatial and temporal trend studies.
